# Sequence Analysis of the Genome of Piscine Orthoreovirus (PRV) Associated with Heart and Skeletal Muscle Inflammation (HSMI) in Atlantic Salmon (*Salmo salar*)

**DOI:** 10.1371/journal.pone.0070075

**Published:** 2013-07-29

**Authors:** Turhan Markussen, Maria K. Dahle, Torstein Tengs, Marie Løvoll, Øystein W. Finstad, Christer R. Wiik-Nielsen, Søren Grove, Silje Lauksund, Børre Robertsen, Espen Rimstad

**Affiliations:** 1 Department of Laboratory Services, National Veterinary Institute, Oslo, Norway; 2 Norwegian College of Fishery Science, University of Tromsø, Tromsø, Norway; 3 Department of Food Safety and Infection Biology, Norwegian School of Veterinary Science, Oslo, Norway; INRA, France

## Abstract

Piscine orthoreovirus (PRV) is associated with heart- and skeletal muscle inflammation (HSMI) of farmed Atlantic salmon (*Salmo salar*). We have performed detailed sequence analysis of the PRV genome with focus on putative encoded proteins, compared with prototype strains from mammalian (MRV T3D)- and avian orthoreoviruses (ARV-138), and aquareovirus (GCRV-873). Amino acid identities were low for most gene segments but detailed sequence analysis showed that many protein motifs or key amino acid residues known to be central to protein function are conserved for most PRV proteins. For M-class proteins this included a proline residue in μ2 which, for MRV, has been shown to play a key role in both the formation and structural organization of virus inclusion bodies, and affect interferon-β signaling and induction of myocarditis. Predicted structural similarities in the inner core-forming proteins λ1 and σ2 suggest a conserved core structure. In contrast, low amino acid identities in the predicted PRV surface proteins μ1, σ1 and σ3 suggested differences regarding cellular interactions between the reovirus genera. However, for σ1, amino acid residues central for MRV binding to sialic acids, and cleavage- and myristoylation sites in μ1 required for endosomal membrane penetration during infection are partially or wholly conserved in the homologous PRV proteins. In PRV σ3 the only conserved element found was a zinc finger motif. We provide evidence that the S1 segment encoding σ3 also encodes a 124 aa (p13) protein, which appears to be localized to intracellular Golgi-like structures. The S2 and L2 gene segments are also potentially polycistronic, predicted to encode a 71 aa- (p8) and a 98 aa (p11) protein, respectively. It is concluded that PRV has more properties in common with orthoreoviruses than with aquareoviruses.

## Introduction

Piscine orthoreovirus (PRV) is associated with heart and skeletal muscle inflammation (HSMI) of farmed Atlantic salmon (*Salmo salar*) [1]. Infectious viral diseases are prevalent in farmed fish, and HSMI is an example of an emerging disease in the intensive farming of Atlantic salmon. HSMI is usually observed 5–9 months after transfer of the fish from the freshwater stage to seawater grow out areas [2] and is characterized by inflammation of the epi-, endo- and myocard and of the red skeletal muscle. The majority of the fish in an affected cage will show lesions in the heart and the cumulative mortality may reach 20% [2]. The virus has not successfully been cultivated continuously in cell cultures, although PRV harvested from two week cultures in GF-1 cells have been used for challenge experiments [3]. The PRV genome was mapped by high-throughput pyrosequencing of material from diseased fish and found to consist of 10 dsRNA segments [1]. By the use of real time RT-PCR it has been shown that PRV is widely distributed among both farmed and wild Atlantic salmon [1], [4]. The sizes of the genomic segments are distributed in the classical orthoreoviral groups L1–3, M1–3 and S1–4. The S1 and S2 segments are possibly bicistronic having accessory small putative open reading frames.

The 3′-terminal nucleotide sequence (UCAUC-3′) in the PRV gene segments is conserved and identical to both orthoreoviruses and aquareoviruses [1], [5], [6]. On the other hand, the 5′-terminal nucleotide sequence (5′-GAUAAA/U) of PRV is unique, as are the analogue sequences for each of the individual species within the *Orthoreovirus* genus (MRV, ARV, Nelson Bay, Baboon and Reptilian orthoreoviruses) and of those species in the *Aquareovirus* genus (Aquareovirus A and C) for which the 5′-end sequences are known [5], [7].

Based upon phylogenetic analysis a common evolutionary origin of the genera *Aquareovirus* and *Orthoreovirus* has been revealed [5]. Phylogenetic analyses performed separately for each PRV segment has shown that this virus branches off the common root of the Aquareovirus and Orthoreovirus genera which could indicate that PRV may represent a genetic new lineage, divergent from other reovirus genera [1]. To our knowledge, aquareoviruses and orthoreoviruses, with an amino acid identity of 42% in the dsRNA-dependent RNA polymerase (RdRp), are the only reovirus genera with identity of more than 30% in the RdRp that are placed in separate genera [5]. The prototype strain mammalian reovirus type 3 Dearing (MRV T3D) was chosen for comparison as this strain is the best studied within the genera. Low amino acid homologies to the MRV proteins λ1, λ2, λ3, µ1, µ2, µ3, σ2 and σNS are found in PRV as well as in the aquareoviruses (AqRV) [1]. AqRV have been isolated from a wide variety of aquatic animals, including molluscs, finfish and crustaceans, while the orthoreoviruses have been found in reptiles, birds and mammals. There is low sequence homology of genes and proteins between the species in genus *Orthoreovirus* indicating a long time divergence [6]. AqRV have 11 genomic segments while both the orthoreoviruses and PRV have 10.

Viral particles of MRV and ARV consist of a double layered protein capsid with inner and outer layers. Studies of MRV indicate that the σ1 protein attaches to cell surface receptors and thus is important for the cell and tissue tropisms [8]–[10]. MRV particles enter the cell through receptor-mediated endocytosis. MAbs directed against the σ1, the σ3 and μ1C outer capsid proteins, and the core spike protein λ2 can neutralize MRV [11].

Following endocytosis, the outer capsid undergoes proteolysis within the acidic compartment of the endosomes, resulting in the removal of σ3 and cleavage of µ1 to µ1C and µ1N [12], [13]. The resulting intermediate subviral particles (ISVPs) penetrate the endosomal lipid bilayer, probably through the action of exposed hydrophobic parts of the cleaved µ1 protein [14]–[17]. This makes endosomal membrane penetration possible and is followed by cytoplasmic release of transcriptionally active viral cores [18], [19]. Inside the cores, full-length capped but non-polyadenylated viral mRNAs are made. Cap formation requires the sequential activity of polynucleotide phosphohydrolase, guanylyltransferase and methyltransferase [20]. The λ1 protein functions as helicase and triphosphatase, λ2 as the guanylyltransferase and λ3 is the RNA polymerase [21]–[25]. *S*-adenosyl-L-methionine (SAM) is the substrate (methyl donor) for the methylation of the type 1 cap mediated by λ2 [24], [26]–[29]. The transcripts act as templates for both translation and replication of viral genomic dsRNA [30].

The present study was performed to compare the properties of the putative PRV proteins to analogues of the orthoreoviruses MRV, ARV and the aquareovirus grass carp reovirus (GCRV). Through *in silico* analysis, 10 deduced amino acid sequences of PRV proteins were assigned. It was concluded that PRV is more related to the genus *Orthoreovirus* than to the *Aquareovirus*.

## Results

### Genome Organization

**Figure 1:**
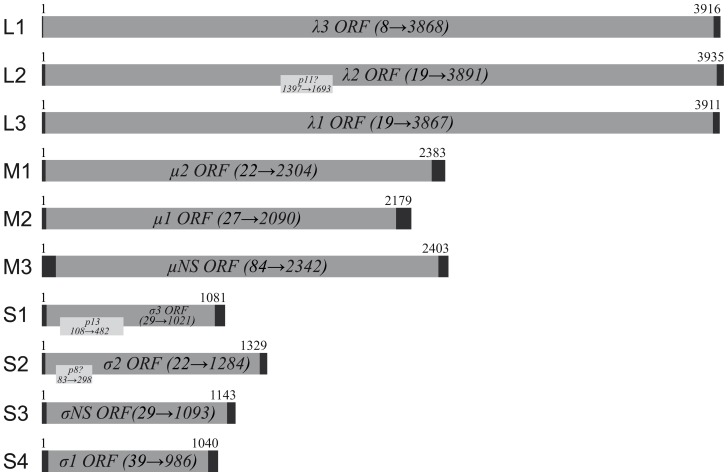
The PRV genome. Gene segments are assigned according to mammalian reoviruses. Open reading frames (ORFs) and putative encoded proteins are indicated by regions in grey, with start and end positions indicated. Non-translated regions (UTR’s) at gene segment ends are shown in black. Gene segments L2, S1 and S2 are possibly polycistronic.

The PRV genome consists of 10 segments containing at least 10, but has possibly 13 ORFs or more. The genome has a length of 23320 nt and a GC content of 47%. For both orthoreoviruses and aquareoviruses the ultimate nucleotides from each end are inverted complements [5], [31]–[34]. The length of the 5′-UTRs was shortest for the L segments, 7–18 nucleotides, while M3 and S4 had the longest with 84 and 38 nucleotides, respectively (**Figure 1**). The length of the 3′-UTRs varied between 44–89 nucleotides, the longest were found in the M segments (**Figure 1**). Segment comparison showed that PRV 5′-UTRs were on average significantly shorter than the 3′-UTRs. In PRV, the three ultimate nucleotides in 5′- and 3′-end of each segment are inverted complements. RNA secondary structure predictions using mFold version 2.3 [35] performed at 15°C of the 5′- and 3′- UTRs of mRNA from each genomic segment was assayed using energy minimization criteria. The predictions were panhandle structures, but the last 3′-end nucleotides were not a part of the stem structures. For segment S2, however, the prediction was not a panhandle structure (data not shown).

The PRV genome segments except for S1 and S4 were assigned according to the assignment used for MRV (**Table 1**). Hence, for example, the PRV segment that encodes the core protein λ2 is called L2, although it is slightly longer than the other L segments. Based upon sequence homology to MRV and ARV in particular, eight of the deduced translation products are assumed structural proteins. For PRV, the S1, S2 and possibly L2 gene segments have additional internal open reading frames (ORFs) in addition to the σ3, σ2 and λ2-encoding ORFs (**Table 1** **,** **Figure 1**).

**Table 1:** Proteins encoded by the PRV genome and functional properties as predicted from comparative studies with selected reovirus prototype strains.

PRV encoded proteins^a^					MRV T3D^b^	ARV-138	GCRV-873^c^
Segment	Protein name	Length (aa)	Theoretical weight (kDa)	Predicted location in virionand functional properties	Protein (length, aa)	Protein (segment number)	Protein (segment number)
						(length, aa)	(length, aa)
L1	λ3	1286	144.2	RNA-dependent RNA polymerase	λ3 (1267)	λB (L2) (1259)	VP2 (S2) (1274)
L2	λ2, p11?	1290	143.7	Guanylyltransferase, methyltransferase	λ2 (1289)	λC (L3) (1285)	VP1 (S1) (1299)
				p11: hypothetical protein			
L3	λ1	1282	141.5	Helicase, NTPase, RNA triphosphatase	λ1 (1275)	λA (L1) (1293)	VP3 (S3) (1214)
M1	µ2	760	86.0	NTPase, RNA triphosphatase, RNA binding	μ2 (736)	µA (M1) (732)	VP5 (S5) (728)
M2	µ1	687	74.2	Outer capsid protein, membrane penetration	μ1 (708),	µB (M2) (676),	VP4 (S6) (648)
					μ1N (42), μ1C (666)	µBN (42), µBC (634)	
M3	µNS	752	83.5	Non-structural protein,	µNS (721),	µNS (M3) (635),	NS80 (S4) (742)
				central in virus inclusion formation	µNSC (681)	µNSC, µNSN	
S1	σ3, p13	330, 124	37.0, 13.0	σ3: outer capsid protein, zinc metalloprotein	σ3 (S4) (365)	σB (S3) (367)	VP7 (S10) (276),
				p13: cytotoxic, integral membrane protein^d^			NS26 (S11) (244)
S2	σ2, p8?	420, 71	45.9, 8.1	σ2: Inner capsid protein, RNA binding	σ2 (S2) (418)	σA (S2) (416)	VP6 (S8) (412)
				p8: hypothetical protein			
S3	σNS	354	39.1	Non-structural protein,	σNS (S3) (366)	σNS (S4) (367)	NS38 (S9) (352)
				involved in virus inclusion formation			
S4	σ1	315	34.6	Cell attachment protein	σ1 (S1) (455),	σC (S1) (326),	NS31 (S7) (274),
					σ1s (S1) (120)	p10 (S1) (98),	NS16 (S7) (146)
						p17 (S1) (146)	

a L1-M3 PRV gene segments are annotated according to mammalian reoviruses (MRV). PRV L1 has been changed to L3, and vice versa, compared to that suggested by [1]. PRV S-class gene segments are annotated according to [1]. For mammalian reovirus (MRV), avian orthoreovirus (ARV) and grass carp reovirus (GCRV) several proteins are produced from alternative reading frames or by post-translational proteolytic cleavage. In the latter case, if the exact cleavage site is known, the lengths of both proteolytic fragments are included in the table.

b T3D = Type 3 Dearing strain.

c GCRV contains an eleventh genomic segment which encodes a non-structural protein, NS26. VP7 is homologues to σ3/σB.

d Cytotoxic, nonfusogenic integral membrane protein [96].

### L-class Gene Segments

#### PRV gene segment L1 is predicted to encode the λ3 protein (Table 1, Figure 1)

This is the virus’ RNA-dependent RNA polymerase (RdRp) responsible for viral transcription and replication. It displays the highest amino acid sequence similarity to MRV, ARV and GCRV (**Table 2**). Multiple sequence alignment of PRV λ3 with the corresponding protein sequences from MRV, ARV and GCRV revealed that important polymerase motifs are also conserved in the PRV protein (**Figure S1**). Comparing with detailed structure-function analysis made of MRV T3D λ3 [36], we found high conservation particularly in the catalytic core (aa 350–900), and of specific amino acids predicted to be responsible for interaction with the RNA template, NTP and the 5′-cap. The previously described catalytic motifs I, II, III and F1–F3 could easily be identified [37]–[40], including the universal RdRp *GDD* motif (within motif III) involved in transcriptional initiation [41]–[43]. The N- and C-terminal domains of the polymerase, predicted to be involved in interaction with the capping protein λ2 and RNA helicase λ1 for MRV, are somewhat less conserved. This is line with a lower sequence conservation of the other PRV λ proteins.

**Table 2:** Percentage amino acid identity among all ungapped positions between pairs; predicted PRV proteins and the homologues proteins from three reovirus prototype strains.

	PRV	PRV	PRV	PRV	PRV	PRV	PRV	PRV	PRV	PRV
	λ3	λ2	λ1	µ2	µ1	µNS	σ3	σ2	σNS	σ1
MRV T3D^a^	43	23	32	20	28	17	13	20	20	21^d^
ARV-138^b^	44	22	31	21	27	17	15	20	18	14
GCRV-873^c^	38	25	31	21	24	13	15	17	13	f-

a,b,c Ref. Table 1 for gene segment annotations and names of homologues proteins in MRV, ARV and GCRV. Identity values are from separate pairwise alignments of the protein sequences.

d Value from a manually adjusted pairwise alignment of the two proteins.

f GCRV does not appear to have a cell attachment protein homologue to σ1/σC.

#### PRV gene segment L2 encodes the λ2 protein (Table 1, Figure 1)

In MRV and ARV (λC) this is the capping enzyme, the main contributor when generating the 5′-terminal cap, on virally encoded mRNAs [24], [26]–[29]. The MRV- and ARV proteins contain both the guanylyltransferase and methyltransferase activities necessary for the generation of this type 1 cap structure [24], [27], [29], [44], [45]. For PRV, multiple sequence alignment reveals relatively low amino acid identities to the corresponding proteins in MRV, ARV and GCRV, although key amino acid residues and important functional domains are highly conserved (**Table 2** **, Figure S2**). These include residues K_190_ which is essential for autoguanylation, H_223_ and H_232_ which are essential for guanylyltransferase activity and the *S*-adenosyl-L-methionine (SAM) binding pocket [44]–[47]. K_171_, a significant but not essential contributor for autoguanylation [46], is not conserved in PRV at this position although a KY motif sits just two positions downstream. A region containing an ATP/GTP binding site in the MRV protein [45] is also highly conserved in all four homologues proteins**.** Also, a hypersensitive cleavage site has been identified when using recombinant MRV λ2 and ARV λC [44], [46]. In the PRV protein, the two amino acids on either side of this cleavage site are identical to those in ARV. Secondary structure predictions using PSIPRED v3.0 [48], [49] suggests that this site resides in a predicted random coil region shortly after a predicted strand region**,** perhaps constituting an exposed region highly susceptible to cleavage by cellular proteases (not shown). Additionally, the L2 gene segment may be polycistronic (**Table 1** **,** **Figure 1**).

#### PRV gene segment L3 is predicted to encode the λ1 protein (Table 1, Figure 1)

This is the core capsid shell homologue of MRV λ1, ARV λA and GCRV VP3 [50]–[52]. Multiple sequence alignment showed higher amino acid identities between PRV and the three prototype strains than observed for λ2 but lower than for λ3 (**Table 2** **, Figure S3**). Key functional residues or domains are conserved in PRV λ1. A CCHH zinc-finger motif in particular, conserved in the MRV, ARV and GCRV proteins and shown to bind zinc in core crystal structure analysis [51], [53], [54], is also conserved in PRV λ1 (**Figure S3**). For MRV, the ∼500 residue N-terminal region has been shown to exhibit triphosphate phosphohydrolase (NTPase), helicase, and RNA triphosphatase activities while the ∼200 residue N-terminal parts, a region predominantly hydrophilic, is assumed to be responsible for the dsRNA binding property of λ1 [21], [23], [52], [55]–[58]. For PRV, as for the homologous proteins from the selected reovirus prototypes, a predominantly N-terminal RNA/DNA binding region was predicted using BindN [59] (data not shown).

### M-class Gene Segments

#### PRV gene segment M1 encodes the μ2 protein (Table 1, Figure 1)

Compared to the proteins encoded by the L-class gene segments, PRV μ2 has a lower amino acid sequence identity with the homologous proteins in the MRV, ARV and GCRV (**Table 2** **, Figure S4**). In MRV, μ2 is a minor structural protein, binds both ssRNA and dsRNA independent of sequence, and possibly acts as a co-factor or subunit of the viral RNA polymerase [30], [60], [61]. In addition, both MRV μ2 and ARV µA display NTPase- and RTPase activities [62]–[64]. In MRV μ2, the two lysines K_415_ and K_419_ in the region A_411_VLPKGSFKS_420_, have been shown to be essential for NTPase activity [63]. Both these lysines are conserved in all four homologous proteins (**Figure S4**). A second putative nucleotide binding motif, D_446_EVG_449_ [65], is only partially conserved. The P_208_ in MRV T3D has also recently been shown to be a determinant of type I IFN antagonism and a modulator of induction of myocarditis in neonatal mice [69]. P_208_ is conserved in all four proteins.

#### PRV gene segment M2 encodes the μ1 protein (Table 1, Figure 1)

This is the homologue of the major outer capsid protein of MRV [54], [70], [71]. In ARV and GCRV the homologous proteins are called µB and VP4, respectively [5], [50]. The highest amino acid sequence identity was found towards the homologous proteins in MRV and ARV, somewhat lower for GCRV (**Table 2**). Multiple sequence alignment showed that the N-terminal sequence parts of the four proteins display higher conservation compared to the rest of the protein (**Figure S5**). A post-translational autolytic cleavage site in the MRV protein between N_42_ and P_43_, which produces a small N-terminal- (μ1N) and a larger C-terminal fragment (μ1C), required for MRV endosomal membrane penetration and infection, is conserved in all four proteins [70], [71]. The cleavage of MRV μ1 during infection is dependent upon N-myristoylation at G_2_, as well as binding to the S1 gene product σ3 (see below) [72]. Similarly, ARV µB is also myristoylated at G_2_ and post-translationally cleaved, with µB and its cleavage product µBC associating with σB [68], [73]. The G_2_-residue is conserved in all four proteins (**Figure S5**). These two processes, myristoylation and cleavage, are believed to be crucial for membrane penetration with μ1N as the principal mediator [17], [74], [75] (see Figure S5 legends for more details).

#### PRV gene segment M3 encodes the non-structural µNS protein (Table 1, Figure 1)

This is the homologous counterpart of MRV and ARV µNS [50], [76], [77]. The ARV µNS protein is significantly shorter compared to the MRV protein, a difference which has been attributed to deletions (**Figure S6**) [78]. In GCRV, the homologous protein is NS80, the product of gene segment 4 [5]. A very low amino acid sequence identity is observed between the PRV protein and the homologous proteins of the three prototype strains (13–17%) (**Table 2**). Regions containing some level of conservation can though be identified (see Figure S6 legends for more details)**.** One such motif, L_711_IDFS_715_, towards the C-terminal end and shown for MRV to be required for the recruitment of clathrin to viral factories [79], is partially conserved in the ARV- and PRV proteins. MRV µNS, ARV µNS and GCRV NS80 have all been predicted to contain two α-helical coiled coils regions in their C-terminal region, which have been shown to be necessary for inclusion formation [86]–[90]. PRV µNS also contains high α-helical content in the C-terminal region and MultiCoil does predict coiled coil formation here although with significant lower probability compared to the MRV-, ARV- and GCRV proteins (not shown).

For both MRV and ARV M3, two products have been reported, the µNS protein representing the full-length isoform. For MRV, the second isoform µNSC (75 kDa) is most likely generated from the second in-frame AUG (Met_41_) [88], [91]. In ARV, post-translational cleavage near the N-terminal region creates µNSN [92]. PRV µNS does not contain a Met_41_, but it does contain a Met_57_, which is also present in MRV (**Figure S6**). Neither the MRV- nor PRV AUG codons encoding Met_57_ comply with the Kozak rule, while the initiation codon for MRV Met_41_ complies partially [93].

### S-class Gene Segments

#### PRV gene segment S1 encodes the major outer capsid protein σ3 (Table 1, Figure 1)

**Figure 2:**
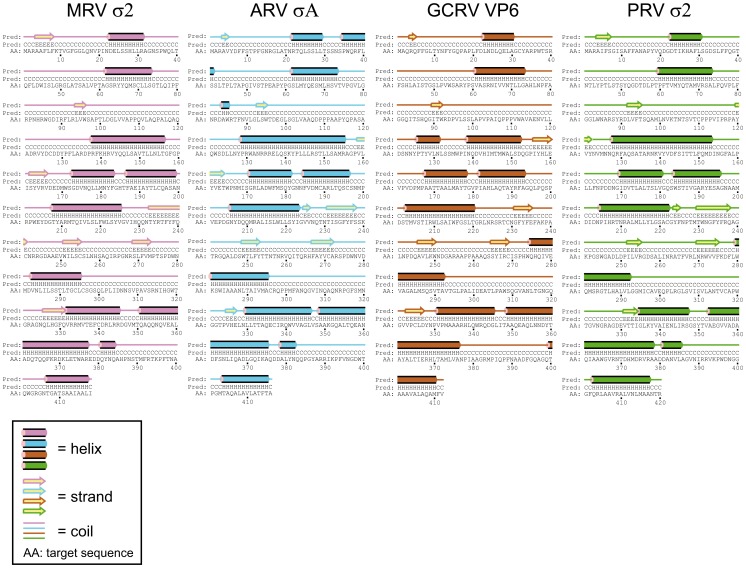
Expression and subcellular localization of the S1-encoded σ3 and p13 proteins in mammalian VERO and salmonid CHSE cells. Immunofluorescent staining of σ3 or p13 (green colour), and staining with the trans-Golgi marker WGA (red colour). Transfected VERO cells (A) and CHSE cells (B) expressing both σ3 and p13 from the large S1 ORF (upper panels), and p13 expression from the S1 internal ORF (lower panels). Nuclei are stained with DAPI (blue colour). Yellow colour indicates colocalization of p13 and WGA. Non-transfected cells stained with WGA and anti-p13 serum was used as controls (CTRL).

In MRV, ARV and GCRV the major outer capsid proteins are encoded by gene segment S4 (σ3), S3 (σB) and segment 10 (VP7), respectively [5], [76], [77], [94], [95]. PRV σ3 was recently determined to be encoded by the second smallest S-class gene segment [96]. Although amino acid identities between the PRV protein and that of the reovirus prototype strains used are very low and in the range of non-related proteins (**Table 2**), a Zn-finger motif is evolutionary conserved in all four proteins (**Figure S7**) [96], [97]. Expression of σ3 in mammalian VERO cells at 37°C and salmonid CHSE cells at 20°C demonstrated that the protein was primarily cytoplasmic. In CHSE cells, green fluorescence was observed diffusely throughout the cytoplasm while in VERO cells the protein seemed to form large inclusions (**Figures 2a, b**).

#### PRV gene segment S2 encodes the core clamp protein σ2 (Table 1, Figure 1)

**Figure 3:**
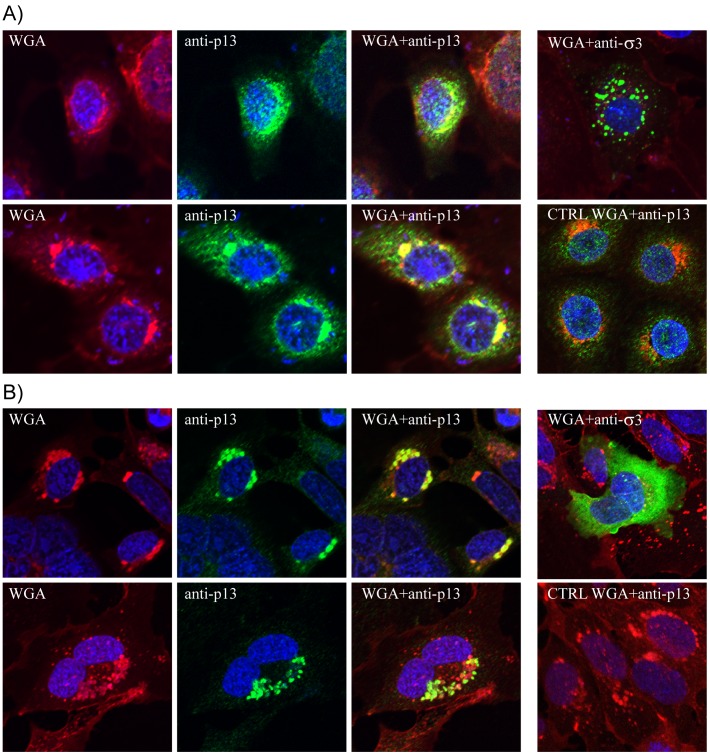
PSIPRED secondary structure predictions of PRV σ2 (green) and the homologous proteins from the reovirus prototype strains MRV T3D (σ2, pink), ARV-138 (σA, blue) and GCRV-873 (VP6, brown).

In MRV, ARV and GCRV the homologues proteins are σ2, σA and VP6, respectively [5], [54], [76], [77], [98], [99]. PRV S2 is the largest S-class gene segment, and possibly bicistronic (**Table 1** **,** **Figure 1**). Multiple sequence alignment of the four σ2/σA/VP6 proteins show overall low amino acid identities (**Table 2**, **Figure S8**). Still, between MRV cores and ARV σA (sharing 29% amino acid identity), comparisons of crystal structure data have shown a highly similar overall topology, including higher α-helical content in their C-terminal regions [54], [100]–[102]. For PRV σ2, PSIPRED [48], [49] also predicts a high α-helical content in its C-terminal region (**Figure 3**). In fact, comparing the predicted secondary structure profiles between all four reovirus proteins reveals a remarkable conservation of secondary structure (**Figure 3**), providing strong support that this gene segment encodes σ2. In further support for the correct annotation of PRV σ2, the predicted pI of the PRV protein is close to that of the MRV- and ARV proteins [96].

#### PRV gene segment S3 encodes the non-structural σNS protein (Table 1, Figure 1)

In MRV and ARV, σNS is encoded by gene segment S3 and S4 respectively, while in GCRV the homologues protein, NS38, is encoded by segment 9 [5], [50], [103]. Similar to the three prototype strains, PRV S3 also contains a single ORF, encoding a protein with size close to that of σNS/NS38 (**Table 1** **,** **Figure 1**). Multiple sequence alignment with these four protein sequences reveals overall very low amino acid sequence identities, not only to the PRV protein but between the three prototype strains as well (**Table 2** **, Figure S9**). In addition, PSIPRED suggests some level of protein structure conservation between PRV σNS and the proteins from the three prototype strains (not shown). Furthermore, the predicted pI of the putative PRV σNS protein shows generally more closeness to the homologous proteins in MRV, ARV and GCRV compared to the other three major PRV S-class proteins [96]. Taken together this suggests that a correct assignment of PRV σNS has been made.

MRV σNS has been detected in both the nucleus and cytoplasm of infected and transfected cells, with the former being linked to its nucleic acid binding capability [85], [103], [104]. PSORTII does not predict the presence of nuclear localization signals in the PRV protein (not shown). But, within regions of the alignment displaying somewhat higher level of conservation, NetNES 1.1 [105] does predict the presence of NESs in all four proteins (**Figure S9**).

#### The major gene product of PRV gene segment S4 is the σ1 cell-attachment protein (Table 1, Figure 1)

In MRV and ARV, the cell attachment proteins σ1 and σC are the major gene products from the bicistronic and tricistronic S1 gene segments, respectively [10], [106]–[109] (**Table 1**). The aquareovirus GCRV does not seem to encode a homologue of the orthoreovirus cell attachment proteins [99], [110]. Rather, GCRV gene segment 7 encodes two non-structural proteins (**Table 1**) [5].

**Figure 4:**
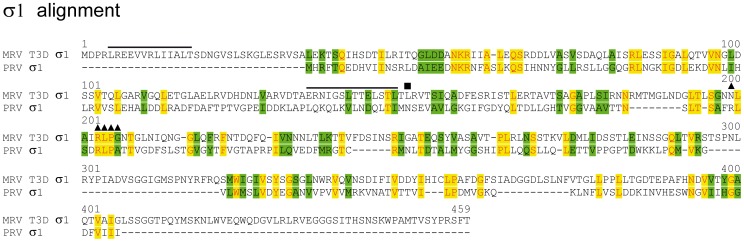
Multiple sequence alignment of PRV σ1 with MRV T3D σ1. Black lines represent putative nuclear export signals (NEP) in MRV and PRV, respectively, as predicted by NetNes 1.1. ▪ = L_149_ in the MRV protein involved in a second predicted NES. ▴ = residues in the MRV protein involved in binding to sialic acid residues. The alignment has been manually adjusted.

Multiple sequence alignment with PRV σ1 and MRV σ1/ARV σC shows amino acid identities in the range of 14–21%, depending on the degree of manual adjustment of the alignment (**Table 2** **,** **Figure 4**; only PRV and MRV aligned). Annotation of PRV σ1, based on the high probability presence of predicted α-helical coiled-coil structure(s) in the N-terminal region of the protein, was recently determined to be similar to that of MRV σ1 and ARV σC (**Figure S10**) [96], [111]–[114]. Secondary structure predictions using PSIPRED provides additional support for a PRV σ1 N-terminal region dominated by α-helixes (not shown). For the GCRV NS31 protein, on the other hand, predicted secondary structure profiles are very different from that of the three other proteins (not shown), with no predicted coil structures (**Figure S10**).

The MRV σ1 protein can broadly be divided into three distinct domains, the N-terminal tail which partially inserts into the virion, the body which contains the region that binds to sialic acids, and the C-terminal head domain, which binds the receptor junctional adhesion-molecule-A (JAM-A) [115]–[119]. For the MRV T3D strain, σ1 binds to α-linked sialic acids [120], [121]. Sequence- and structure analyses of MRV variants have indicated that amino acids in positions 198, 202–205 are involved in binding to sialic acids [122]–[124]. The alignment in **Figure 4** suggests that several of these MRV σ1 residues may be conserved in PRV, but not in the ARV protein (not shown). In fact, these residues are less conserved in the MRV T1L and T2J serotypes compared to PRV σ1 (not shown). Finally, the predicted isoelectric point (pI) for this putative PRV protein lies in the acidic range, as do MRV σ1 and ARV σC [96]. All together, we provide additional support that this protein, encoded by the smallest PRV S-class gene segment, is the cell attachment protein homologous to those of other orthoreoviruses.

### Accessory ORFs

PRV gene segment S1 is bicistronic and has an internal ORF encoding a 124 aa protein (p13) (**Table 1** **,** **Figure 1**). This protein is expressed from the σ3 ORF when transfected in VERO and CHSE cells, as determined by immunofluorescent staining using anti-p13 serum, where it colocalizes with the trans-Golgi marker WGA (**Figures 2a, b**). Colocalization of p13 with this marker was also seen following transfection with an expression plasmid containing only the p13 ORF (**Figures 2a, b**). The p13 protein was originally assumed to be a fusion-associated small transmembrane (FAST) protein capable of inducing cell-cell fusion and syncytium formation, but was recently determined to encode a nonfusogenic integral membrane protein with cytotoxic properties [1], [96]. Scanning the PRV genome for potential accessory ORFs revealed several potential candidates. Both S2 and L2 are putative polycistronic gene segments, where the former has the potential to encode a second 71 aa hypothetical protein (p8) and the latter contains several smaller ORFs with AUG- or non-canonical GUG/CUG start codons that could encode hypothetical proteins of sizes ranging from 55 to 135 aa. The possibility of translation initiation from non-canonical start codons should not be excluded, as has been shown for the AtSRV p22 protein which is produced from a noncanonical CUG translation start codon in segment 7 [125]. Besides the λ2-encoding ORF, the largest L2 ORF containing an AUG translation initiation codon could encode a small protein of 98 aa (p11) (**Table 1** **,** **Figure 1**). None of these putative gene products display any apparent sequence similarities towards the additional gene products from MRV S1 (σ1s), ARV S1 (p10, p17) or GCRV segment 7 (NS16) (not shown). ARV p10 and GCRV NS16 have been shown to be FAST proteins capable of inducing cell-cell fusion and syncytium formation [126], [127]. No sequence similarities to other known reovirus FAST proteins, such as Atlantic salmon reovirus (AtSRV p22), Turbot reovirus (SMReV p22), Baboon reovirus (BRV p15), Reptilian reovirus (RRV p14), Broome virus (BroV p13) and Nelson Bay reovirus (NBV p10) was observed either (not shown) [7], [125], [127]–[130]. Low or absence of sequence similarities at the primary amino acid sequence level is though a common trait among reovirus FAST proteins [131]. ProtScale hydrophobicity plots does indicate the presence of structural motifs in both these hypothetical proteins characteristic of FAST proteins (transmembrane domain (TMD), hydrophobic patch (HP), basic region (PB)), with patterns similar to ARV p10, and less similar to GCRV NS16 [127], [131] (**Figure S11**). The polybasic region is though shorter and less defined in the PRV proteins. Still, both are predominantly hydrophobic and have high predicted pI’s of 8,81 (p8) and 9,38 (p11) which is similar to that of other known FAST proteins (8.80–9.84). Also, the AUG translation initiation codon in the p8 gene complies with Kozak [93]. In contrast, the p11 AUG translation initiation codon deviates from Kozak in all positions. The present study may provide an example of deviations from Kozak consensus, as the AUG translation initiation codon in the PRV p13 ORF deviates from Kozak consensus both at positions −3 and+4. All known FAST proteins are also modified either by palmitoylation or myristoylation [127]. No myristoylation- or S-palmitoylation sites were predicted for the hypothetical p8 protein. In contrast, S-palmitoylation sites were predicted for p11, at C_13_ and C_14_. Assuming a N_exo_/C_cyt_ surface membrane topology for p11, the location of these sites differ from the S-palmitoylation sites in ARV- and NBV p10, which are located between their transmembrane and polybasic regions (**Figure S11**) [127]. Also, reovirus FAST proteins may contain N-glycosylation signals in their sequence [129], [130] **.** Two potential N-glycosylation sites were predicted in p11, at position 8 and 66. None were predicted in p8. A distinct polyproline motif, common in some reovirus FAST proteins, was not present in p8 and p11. However, in the latter, although not clustered together, there are three proline residues towards the C-terminal end of which two flank the basic region.

Whether either of these two putative PRV proteins are homologues to other known reovirus FAST proteins or not, information which would enable a better classification of PRV, will require further experimental investigations. It should though be noted that syncytia are not common histopathological findings in HSMI diseased fish [2], which would suggest that PRV is non-fusogenic. Therefore, the possibility that the hypothetical accessory PRV proteins display functional properties similar to the σ1s and p17 proteins encoded by the bicistronic genes in MRV and ARV, respectively, should be considered.

## Discussion

In the present study we have performed detailed comparative sequence analysis of the non-translated (UTR) regions and the putative proteins encoded by the PRV genome to prototype strains from mammalian and avian orthoreoviruses, and one aquareovirus. The results suggest that the PRV genome encodes at least 10 proteins, but it may also contain up to 13 or more ORFs.

In general, amino acid identities between PRV and the three prototype strains were low for most gene segments, highest for the L-class gene segment encoding the RdRp. Functional constraints often cause viral core proteins to remain more conserved than the outer capsid proteins, as illustrated by the conserved structural motifs predicted for PRV proteins λ1 and σ2. In the latter case, a remarkable strong conservation of protein secondary structure across genus lines of orthoreoviruses and aquareoviruses, was predicted. This illustrates conservation of secondary structure over primary sequence, reflecting the importance of structural features for function(s), where even minor alterations would be deleterious. Furthermore, residues important for the enzymatic actions of reovirus proteins are to a larger extent more conserved than other residues, as exemplified by the PRV λ3 and λ2 proteins. Examples of conserved amino acid residues include the two lysines essential for the NTPase activity of µ2 [63] and the post-translational autolytic cleavage- and myristoylation sites in µ1 [68], [70], [71], [73]. The latter strongly suggests that analogues mechanisms are involved in membrane penetration by this PRV protein as in orthoreo- and aquareoviruses. The amino acid identities between PRV and the prototype strains for the µNS, σ1 and σ3 proteins were very low or similar to that of non-related proteins (<20%) [31]. Sequence analysis did, though, reveal protein motifs or key amino acid residues linked to important protein functions are conserved also in PRV, particularly to those of MRV and ARV.

For the µNS proteins (NS80 in GCRV), the limited sequence similarities may be linked to its multifunctionality. Together with σNS (NS38 in GCRV) they are central in recruitment of core proteins and forming viral factories, sequence independent binding of ssRNA, and vital in RNA packaging and replication [66], [67], [68], [80]–[87], [103], [132]. Virus proteins that make up the core have evolved over time due to selection pressures and thus the resulting rate of change in multifunctional proteins would be higher by comparison, resulting in only rudimentary similarities being left at the primary sequence level, although important structural features may be more conserved. Whether the PRV µNS gene also produces two gene products as the MRV protein does, or whether post-translational cleavage is involved as in ARV µNS, could not be determined from sequence information alone.

Several proteins encoded by reoviruses have been shown, or are suggested, to exhibit type I IFN antagonistic properties, including MRV µ2, ARV σA and MRV σ3 [133]–[137]. A previously established reporter gene system [138] was used to investigate whether expression of PRV μ2, σ2 and σ3 could have an antagonistic effect on the type I IFN response in a salmonid cell line (not shown). The reporter constructs use a salmon minimal type I IFN (IFNa1) promotor [139] and an interferon-stimulated response element (ISRE)-reporter (Agilent technologies). Transfection method and activation of type I IFN response was performed as previously described [138], [139]. No effect could be detected by any of the three viral proteins, neither on IFNa1- or ISRE induction.

The monocistronic S4 gene segment was determined to encode the PRV cell attachment protein σ1, the finding supported by predicted pI’s but more importantly, by structural motifs common to both MRV σ1 and ARV σC, as was recently also described by others [96]. Several amino acid residues in MRV σ1 shown to bind to sialic acids may also be conserved in the PRV protein, which might suggest that PRV utilizes a receptor mediated uptake mechanism involving similar but not identical sialic acid structures.

PRV σ3 expression in mammalian VERO cells at 37°C and in salmonid CHSE cells at 20°C showed a predominantly cytoplasmic localization, although the staining pattern differed in the two cell lines. The punctuated staining pattern observed in the VERO cells may be linked to aberrant folding of the protein at the higher temperature.

FAST proteins have been reported from the *Orthoreovirus* and *Aquareovirus* genera [131]. In contrast to fusogenic reoviruses, for which syncytia are commonly registered, syncytia are not common histopathological findings in HSMI diseased fish [2]. Sequence data alone was not sufficient to determine whether the putative p8, p11 or p13 could be a FAST protein. All three proteins contained predicted properties or motifs only partially consistent with FAST proteins. Recently, using an avian cell line cultivated at 37°C, it was shown that p13 is a cytotoxic protein binding to intracellular membranes, and not a FAST protein [96]. Our findings supported this, and we show that p13 was produced from the internal ORF in the σ3 coding sequence, in both VERO- and the CHSE cells, where it colocalizes with a marker for the trans-Golgi network. The colocalization of p13 and this marker was further supported by the transfection assays using an expression plasmid construct containing the p13 ORF only. Transfection studies with expression plasmid constructs containing the p13 ORF performed in both VERO and CHSE cells have failed to induce cell-cell fusion (data not shown). Transfection with expression plasmid constructs containing the putative p8 and p11 ORFs were not performed. The absence of observed fusogenic activity of PRV and lack of induced syncytia after cellular expression of p13 may indicate that PRV is not fusogenic, as also recently described for p13 [96]. It should not be excluded though that efficient cell-cell fusion is dependent upon a second coexpressed PRV protein, as seen for the GCRV NS16 whose activity is enhanced following coexpression with NS26 [126]. Also, it should be considered whether p13 is a functional equivalent to the MRV σ1s or ARV p17 proteins. Here, σ1s has been shown to have a key role in hematogenous dissemination of the virus [140], [141], involved in reovirus-induced apoptosis [142] and in G(2)/M cell cycle arrest [143]. ARV p17 has also been shown to be involved in G(2)/M cell cycle arrest and shutoff of host protein translation [144] and also acts as a nucleocytoplasmic shuttling protein [108], [127], [145]. Further studies are warranted in order to elucidate the function of p13 and the putative proteins encoded by the internal S2- and L2 ORFs regarding their potential functional properties.

For members of the Reoviridae more than 30% amino acid sequence identity of the RdRp is used as indicative of genus affiliation. But there are exceptions to this, the Rotavirus B polymerase is only 22% identical to other rotaviruses [5]. Sequence alignment of the PRV λ3 protein showed several conserved polymerase motifs, and the identities to the RdRps of the orthoreoviruses MRV and ARV, and the aquareovirus GCRV, were well above the 30% limit. However, the amino acid sequence identities between the RdRps from the orthoreoviruses MRV, ARV and the aquareovirus GCRV is above 40% (not shown), which disqualifies the use of this quantitative taxonomic criterion to distinguish between these genera. However, there are differences between orthoreoviruses and aquareoviruses that justify to keep them as separate genera, as listed by Attoui and co-workers [5], like distinct econiches, 10 versus 11 segments, the GC-content of orthoreoviruses is 44–48% while that of aquareoviruses is 52–60%, many orthoreoviruses do not induce syncytia in contrast to the majority of known aquareoviruses, and there is no antigenic relationship between them. Of these criterions PRV has the following in common with the orthoreoviruses: 10 dsRNA segments, 47% GC-content, syncytia is not reported as a common histopathological finding, nor in cell culture where virus isolation has been attempted (personal observation). The only common criterion with aquareoviruses is the econiche, if fish consisting of a large number of heterogenous species from very different environments should be regarded as a single econiche. The antigenic relationships are unknown.

PRV is not the first reovirus with 10 genomic segments that has been described from fish. A virus isolated in Thailand from the striped snakehead fish (Ophicephalus striatus, also known as Channa striata) with epizootic ulcerative syndrome, was also found to contain 10 genomic segments [146]. However, no nucleotide sequences are available for this virus. The lack of serological cross-neutralization activity to other AqRV, and the difference in number of gene segments made the authors conclude that the virus was not a member of the Aquareovirus genus.

To conclude, although it probably is many million years since the most recent common ancestor for PRV and orthoreo- and aquareoviruses existed, we found conserved structural motifs and somewhat less conserved sequence motifs for all 10 PRV genomic segments. All together, the PRV has more properties in common with the *Orthoreovirus* genus than with the *Aquareovirus* and should hence be renamed Piscine orthoreovirus.

## Materials and Methods

### Computer Analyses

GenBank accession numbers for all PRV, MRV T3D, ARV-138 and GCRV-873 sequences used in the present study is shown in **Table S1**. Multiple sequence alignments of protein sequences were performed in AlignX (Vector NTI Advance™ 11 Package, Invitrogen Dynal AS). RNA secondary structure predictions were performed using mFold version 2.3 with the eighty ultimate 5′- and 3′- nucleotides of the mRNAs from each segment as input sequence (http://mfold.rna.albany.edu/?q=mfold/RNA-Folding-Form2.3) [35]. Default parameters were used in the predictions except for temperature, which was set to 15°C. PSIPRED v3.0 was used for predictions of protein secondary structures (http://bioinf.cs.ucl.ac.uk/psipred/). BindN was used to predict putative RNA binding properties of the proteins (http://bioinfo.ggc.org/bindn/) [59]. The NetNES 1.1 server was used to predict putative leucine-rich nuclear export signals (NESs) in the proteins (http://www.cbs.dtu.dk/services/NetNES/) [105]. Multicoil and COILS were used to predict the presence of putative coiled coil regions in proteins (http://groups.csail.mit.edu/cb/multicoil/cgi-bin/multicoil.cgi, http://embnet.vital-it.ch/software/COILS_form.html) [147], [148]. Default settings were used in the predictions except for Multicoil were window size of 21 was used. Prediction of theoretical molecular weights and isoelectric points (pI’s) for putative PRV proteins was performed using the Compute pI/Mw tool available at http://web.expasy.org/compute_pi/. The presence of putative nuclear localization signals (NLS) in PRV proteins was investigated using PSORTII available at http://psort.hgc.jp/form2.html. Prediction of N-terminal myristoylation was performed using the tools available at http://web.expasy.org/myristoylator/and http://mendel.imp.ac.at/myristate/SUPLpredictor.htm. ProtScale, available at http://web.expasy.org/protscale/with the algorithm by Kyte and Doolittle [149], was used to generate hydrophobicity plots for hypothetical PRV proteins encoded by accessory ORFs and the FAST proteins from ARV-138 and GCRV-873. Prediction of S-palmitoylation sites was performed using CSS-Palm 3.0 using the highest threshold setting [150]. Putative N-glycosylation sites in PRV proteins was predicted using the NetNglyc 1.1 server available at http://www.cbs.dtu.dk/services/NetNGlyc/.

### Cloning, Transfection and Immunofluorescence Staining

The samples were collected from fish originating from a natural outbreak of heart and skeletal muscle inflammation (HSMI) in a commercial Atlantic salmon fish farm (MH-050607). The fish were dead, caused by HSMI, when samples were taken. Thus no approval from Institutional Animal Care and Use Committee (IACUC) or ethics committee was necessary. No experiments that involved fish were performed. The PRV σ3 ORF was amplified by RT-PCR from heart/kidney tissue of HSMI-diseased fish, cloned into the pET-100 plasmid vector and expressed in *E. coli* according to the manufacturer’s instructions (Invitrogen). Proteins were purified from SDS-PAGE gels and used to raise PRV σ3 specific polyclonal anti-serum in rabbits. Antibody specificity was confirmed in western blot. The anti-p13 was produced by immunization of one rabbit with 0.5 mg Keyhole limpet hemocyanin (KLH) peptide, p13 ORF aa104–113, per injection (GenScript Corp, Piscataway, NJ). For both antigens the primary immunization was administered with Freund’s complete adjuvant and the following with Freund’s incomplete adjuvant. Antibody specificity to the KLH peptide was confirmed in a dot blot. The PRV σ3 and the p13 ORFs were also cloned in the pcDNA3.1 vector for expression in eukaryotic cells. VERO cells (ATCC®CCL-81™) grown at 37°C were plated on 0.17 mm cover slips in 24 well plates (1×10^5^ cells/well), and transfected the following day in 500 µl Opti-MEM (Invitrogen) added a mixture of 2 µg expression plasmid and 4 µl of Xtremegene transfection reagent (Roche). Chinook salmon embryo (CHSE-214) cells (Sigma-Aldrich) grown at 20°C were transfected using an Amaxa Nucleofector I Device (Lonza). Three million cells were diluted in 100 µl Ingenio Electroporation Solution (Mirus), added 5 µg expression plasmid and transfected using the Amaxa T-20 program, followed by plating on 0.17 mm cover slips in 24 well plates. VERO cells were incubated for 24h and CHSE cells for 72h, after which cells were fixed in 4% formaldehyde and treated with 5 µg/ml Wheat Germ Agglutinin (WGA) Alexa Fluor 555 conjugate (Molecular Probes). Cell were washed with Hank’s balanced salt solution (HBSS) (Sigma-Aldrich**)** and permeabilized with BD Cytofix/Cytoperm (BD Biosciences Pharmingen, CA, USA). Primary antiserum anti-σ3 (1∶1000) or anti-p13 (1∶1000) was added, followed by secondary antibody Alexa Fluor 488-conjugated anti-rabbit IgG (1∶500) (Molecular Probes). Subsequent wash steps were performed with BD Perm/Wash solution (BD Biosciences Pharmingen). The cover slips were mounted using ProLong Gold antifade mounting media containing DAPI for nuclear staining (Molecular Probes), and cells were visualized on a Zeiss LSM710 confocal microscope (Zeiss, Jena, Germany). The fluorochromes DAPI, Alexa Fluor 488 and Alexa Fluor 555 were excited by lasers at 405 nm, 488 nm and 561 nm, respectively.

## Supporting Information

Figure S1 **Multiple sequence alignment of PRV L1 ORF (λ3) with corresponding ORFs from the reovirus prototype strains MRV T3D, ARV-138 and GCRV-873.** RNA-dependent RNA polymerase (RdRp) domains are indicated with the universally conserved GDD domain (in Motif III) boxed.
(TIF)

Figure S2 **Multiple sequence alignment of PRV L2 ORF (λ2) with guanylyltransferases from the reovirus prototype strains MRV T3D, ARV-138 and GCRV-873.** * = lysine residues in MRV essential (K_190_) or significant contributor (K_171_) for autoguanylation in the MRV, ARV and GCRV proteins, ▴ =  conserved histidines essential for guanylyltransferase activity in the MRV protein, ▪ = ATP/GTP binding site motif A in ARV, boxed = *S*-adenosyl-L-methionine (SAM) binding pocket, • = ATP/GTP binding site motif A in MRV, and ↓ = hypersensitive cleavage site in recombinant MRV λ2 and ARV 1733 λC.
(TIF)

Figure S3 **Multiple sequence alignment of PRV L3 ORF (λ1) with the helicase-NTPase/core capsid shell proteins from the reovirus prototype strains MRV T3D, ARV-138 and GCRV-873.** * = conserved CCHH zinc-finger motif.
(TIF)

Figure S4 **Multiple sequence alignment of PRV M1 ORF encoding the μ2 protein with the homologues proteins from the reovirus prototype strains MRV T3D, ARV-138 and GCRV-873.** ▴ =  conserved proline residue suggested to play a key role in the formation and structural organisation of reovirus inclusion bodies, a determinant of type I IFN antagonism and a modulator of myocarditis in neonatal mice. • = leucine vs. phenylananine, a determinant of tissue tropism of MRV μ2 in MDCK cells. ▪ = possible NLS in MRV. Red lines = nucleotide binding/triphosphate phosphohydrolase regions, and * = conserved lysine residues essential for ATPase activity in ARV µA. A nuclear export signal (NES) has been predicted for MRV μ2 (residues 328–335) [151]. NetNES 1.1 predicts a NES in ARV µA in the same region, while in GCRV, L_233_, L_238_ and L_607_ are predicted to participate in a NES, and in PRV L_80_ (numbering according to the GCRV and PRV sequences, respectively) (not shown).
(TIF)

Figure S5 **Multiple sequence alignment of PRV M2 ORF encoding the μ1/µB major outer capsid protein with the homologues proteins from the reovirus prototype strains MRV T3D, ARV-138 and GCRV-873.** * = myristoylation site in the MRV protein. ↓ = post-translational cleavage site producing N- and C-terminal fragment μ1N and μ1C (MRV) or µBN and µBC (ARV). The C-terminal end of the MRV protein is extended by 33 amino acids compared to the homologous proteins in ARV and GCRV. The PRV protein is also extended, by 28 amino acids.
(TIF)

Figure S6 **Multiple sequence alignment of PRV M3 ORF encoding the putative µNS protein aligned with µNS/NS80 proteins from the reovirus prototype strains MRV T3D, ARV-138 and GCRV-873.** ↓ = N-terminal end of second translation product of MRV (µNSC). Met-57, conserved in MRV and PRV is boxed red. * = conserved putative zinc-hook motif crucial in the formation of inclusion-like structures in the MRV protein [86,90,152]. Black lines indicate sequence regions with higher level of conservation with the motif XGXDPX being boxed. In ARV, the larger region forms part of a region that has been shown to be involved in inclusion maturation [86]. Grey solid and dotted lines = coil-coil(s) regions as predicted by MultiCoil (window size: 21, probability cutoff: 0,5). The MRV L_711_IDFS_715_ motif shown to be required for the recruitment of clathrin to viral factories is boxed red.
(TIF)

Figure S7 **Multiple sequence alignment of PRV S1 ORF encoding the major outer capsid σ3 protein with the homologues proteins from the reovirus prototype strains MRV T3D, ARV-138 and GCRV-873.** * = conserved Zn-finger motif.
(TIF)

Figure S8 **Multiple sequence alignment of PRV S2 ORF encoding the inner capsid σ2 protein with the homologues proteins from the reovirus prototype strains MRV T3D σ2, ARV σA and GCRV-873 VP6.** * = R_273_, one of two arginines in ARV σA linked to dsRNA binding and nucleolar localization, conserved in fusogenic orthoreoviruses.
(TIF)

Figure S9 **Multiple sequence alignment of PRV S3 ORF encoding the putative σNS protein aligned with σNS/NS38 proteins from reovirus prototype strains MRV T3D, ARV-138 and GCRV-873.** Solid black lines represent sequence regions of higher conservation containing putative nuclear export signals.
(TIF)

Figure S10 **COILS prediction of coiled coil regions in PRV σ1 compared to that of the reovirus prototype strains MRV T3D, ARV-138 and GCRV-873.** X-axis displays amino acid positions and the y-axis probabilities.
(TIF)

Figure S11 **Hydrophobic characters of the hypothetical PRV proteins p11 and p8 as predicted by ProtScale compared to the FAST proteins from ARV-138 and GCRV-873.** Predictions were performed using the algorithm by Kyte and Doolittle [149] averaged over a window of nine residues. Positive- and negative scores indicate hydrophobic- and hydrophilic amino acids, respectively. TM = transmembrane domains, PB =  polybasic regions and HP = Hydrophobic patch.
(TIF)

Table S1 **Genbank accession numbers for reovirus nucleotide sequences used in the study.**
(DOCX)

## References

[1] Palacios G, Løvoll M, Tengs T, Hornig M, Hutchison S, et al (2010) Heart and skeletal muscle inflammation of farmed salmon is associated with infection with a novel reovirus. PLoS One 5: e11487. 20634888 10.1371/journal.pone.0011487 PMC2901333

[2] Kongtorp RT, Taksdal T, Lyngøy A (2004) Pathology of heart and skeletal muscle inflammation (HSMI) in farmed Atlantic salmon Salmo salar. Dis Aquat Organ 59: 217–224. 15264718 10.3354/dao059217

[3] Mikalsen AB, Haugland Ø, Rode M, Solbakk IT, Evensen Ø (2012) Atlantic salmon reovirus infection causes a CD8 T cell myocarditis in Atlantic salmon (Salmo salar L.). PLoS One 7: e37269. 22693625 10.1371/journal.pone.0037269 PMC3367920

[4] Løvoll M, Wiik-Nielsen J, Grove S, Wiik-Nielsen CR, Kristoffersen AB, et al (2010) A novel totivirus and piscine reovirus (PRV) in Atlantic salmon (Salmo salar) with cardiomyopathy syndrome (CMS). Virol J 7: 309. 21067578 10.1186/1743-422X-7-309 PMC2994541

[5] Attoui H, Fang Q, Mohd JF, Cantaloube JF, Biagini P, et al (2002) Common evolutionary origin of aquareoviruses and orthoreoviruses revealed by genome characterization of Golden shiner reovirus, Grass carp reovirus, Striped bass reovirus and golden ide reovirus (genus Aquareovirus, family Reoviridae). J Gen Virol 83: 1941–1951. 12124458 10.1099/0022-1317-83-8-1941

[6] Duncan R (1999) Extensive sequence divergence and phylogenetic relationships between the fusogenic and nonfusogenic orthoreoviruses: a species proposal. Virology 260: 316–328. 10417266 10.1006/viro.1999.9832

[7] Thalmann CM, Cummins DM, Yu M, Lunt R, Pritchard LI, et al (2010) Broome virus, a new fusogenic Orthoreovirus species isolated from an Australian fruit bat. Virology 402: 26–40. 20350736 10.1016/j.virol.2009.11.048

[8] Weiner HL, Drayna D, Averill DR Jr, Fields BN (1977) Molecular basis of reovirus virulence: role of the S1 gene. Proc Natl Acad Sci U S A 74: 5744–5748. 271999 10.1073/pnas.74.12.5744 PMC431870

[9] Weiner HL, Powers ML, Fields BN (1980) Absolute linkage of virulence and central nervous system cell tropism of reoviruses to viral hemagglutinin. J Infect Dis 141: 609–616. 6989930 10.1093/infdis/141.5.609

[10] Lee PW, Hayes EC, Joklik WK (1981) Protein sigma 1 is the reovirus cell attachment protein. Virology 108: 156–163. 7269235 10.1016/0042-6822(81)90535-3

[11] Tyler KL, Mann MA, Fields BN, Virgin HW (1993) Protective anti-reovirus monoclonal antibodies and their effects on viral pathogenesis. J Virol 67: 3446–3453. 8388508 10.1128/jvi.67.6.3446-3453.1993 PMC237690

[12] Silverstein SC, Astell C, Levin DH, Schonberg M, Acs G (1972) The mechanisms of reovirus uncoating and gene activation in vivo. Virology 47: 797–806. 5012651 10.1016/0042-6822(72)90571-5

[13] Chang CT, Zweerink HJ (1971) Fate of parental reovirus in infected cell. Virology 46: 544–555. 5167655 10.1016/0042-6822(71)90058-4

[14] Lucia-Jandris P, Hooper JW, Fields BN (1993) Reovirus M2 gene is associated with chromium release from mouse L cells. J Virol 67: 5339–5345. 8350400 10.1128/jvi.67.9.5339-5345.1993 PMC237933

[15] Chandran K, Walker SB, Chen Y, Contreras CM, Schiff LA, et al (1999) In vitro recoating of reovirus cores with baculovirus-expressed outer-capsid proteins mu1 and sigma3. J Virol 73: 3941–3950. 10196289 10.1128/jvi.73.5.3941-3950.1999 PMC104172

[16] Sturzenbecker LJ, Nibert M, Furlong D, Fields BN (1987) Intracellular digestion of reovirus particles requires a low pH and is an essential step in the viral infectious cycle. J Virol 61: 2351–2361. 2885424 10.1128/jvi.61.8.2351-2361.1987 PMC255643

[17] Chandran K, Farsetta DL, Nibert ML (2002) Strategy for nonenveloped virus entry: a hydrophobic conformer of the reovirus membrane penetration protein micro 1 mediates membrane disruption. J Virol 76: 9920–9933. 12208969 10.1128/JVI.76.19.9920-9933.2002 PMC136509

[18] Chandran K, Nibert ML (2003) Animal cell invasion by a large nonenveloped virus: reovirus delivers the goods. Trends Microbiol 11: 374–382. 12915095 10.1016/s0966-842x(03)00178-1

[19] Guglielmi KM, Johnson EM, Stehle T, Dermody TS (2006) Attachment and cell entry of mammalian orthoreovirus. Curr Top Microbiol Immunol 309: 1–38. 16909895 10.1007/3-540-30773-7_1

[20] Furuichi Y, Muthukrishnan S, Tomasz J, Shatkin AJ (1976) Mechanism of formation of reovirus mRNA 5′-terminal blocked and methylated sequence, m^7^GpppG^m^pC. J Biol Chem 251: 5043–5053. 821947

[21] Bisaillon M, Bergeron J, Lemay G (1997) Characterization of the nucleoside triphosphate phosphohydrolase and helicase activities of the reovirus lambda1 protein. J Biol Chem 272: 18298–18303. 9218469 10.1074/jbc.272.29.18298

[22] Bisaillon M, Lemay G (1997) Characterization of the reovirus lambda1 protein RNA 5′-triphosphatase activity. J Biol Chem 272: 29954–29957. 9368073 10.1074/jbc.272.47.29954

[23] Noble S, Nibert ML (1997) Characterization of an ATPase activity in reovirus cores and its genetic association with core-shell protein lambda1. J Virol 71: 2182–2191. 9032352 10.1128/jvi.71.3.2182-2191.1997 PMC191325

[24] Cleveland DR, Zarbl H, Millward S (1986) Reovirus guanylyltransferase is L2 gene product lambda 2. J Virol 60: 307–311. 3018296 10.1128/jvi.60.1.307-311.1986 PMC253932

[25] Wiener JR, Joklik WK (1989) The sequences of the reovirus serotype 1, 2, and 3 L1 genome segments and analysis of the mode of divergence of the reovirus serotypes. Virology 169: 194–203. 2922925 10.1016/0042-6822(89)90055-x

[26] Furuichi Y, Morgan M, Muthukrishnan S, Shatkin AJ (1975) Reovirus messenger RNA contains a methylated, blocked 5′-terminal structure: m^7^G(5′)ppp(5′)G^m^pCp-. Proc Natl Acad Sci U S A 72: 362–366. 1054511 10.1073/pnas.72.1.362 PMC432305

[27] Fausnaugh J, Shatkin AJ (1990) Active site localization in a viral mRNA capping enzyme. J Biol Chem 265: 7669–7672. 2159008

[28] Mao ZX, Joklik WK (1991) Isolation and enzymatic characterization of protein lambda 2, the reovirus guanylyltransferase. Virology 185: 377–386. 1656591 10.1016/0042-6822(91)90785-a

[29] Martinez-Costas J, Varela R, Benavente J (1995) Endogenous enzymatic activities of the avian reovirus S1133: identification of the viral capping enzyme. Virology 206: 1017–1026. 7856076 10.1006/viro.1995.1024

[30] Schiff LA, Nibert ML, Tyler KL (2007) Orthoreoviruses and their replication. In: Knipe DM, Howley PM, Griffin DE, Lamb RA, Martin MA et al.., editors. Fields Virology. 5 ed: Lippincott Williams & Wilkins, Philadelphia, PA. 1853–1915.

[31] Mohd JF, Goodwin AE, Belhouchet M, Merry G, Fang Q, et al (2008) Complete characterisation of the American grass carp reovirus genome (genus Aquareovirus: family Reoviridae) reveals an evolutionary link between aquareoviruses and coltiviruses. Virology 373: 310–321. 18191982 10.1016/j.virol.2007.12.006

[32] Chapell JD, Goral MI, Rodgers SE, dePamphilis CW, Dermody TS (1994) Sequence diversity within the reovirus S2 gene: reovirus genes reassort in nature, and their termini are predicted to form a panhandle motif. J Virol 68: 750–756. 8289378 10.1128/jvi.68.2.750-756.1994 PMC236511

[33] Li JK, Keene JD, Scheible PP, Joklik WK (1980) Nature of the 3′-terminal sequences of the plus and minus strands of the S1 gene of reovirus serotypes 1, 2 and 3. Virology 105: 41–51. 6158163 10.1016/0042-6822(80)90154-3

[34] Zou S, Brown EG (1992) Identification of sequence elements containing signals for replication and encapsidation of the reovirus M1 genome segment. Virology 186: 377–388. 1733095 10.1016/0042-6822(92)90003-8

[35] Zuker M (2003) Mfold web server for nucleic acid folding and hybridization prediction. Nucleic Acids Res 31: 3406–3415. 12824337 10.1093/nar/gkg595 PMC169194

[36] Tao Y, Farsetta DL, Nibert ML, Harrison SC (2002) RNA synthesis in a cage–structural studies of reovirus polymerase lambda3. Cell 111: 733–745. 12464184 10.1016/s0092-8674(02)01110-8

[37] Bisaillon M, Lemay G (1999) Computational sequence analysis of mammalian reovirus proteins. Virus Genes 18: 13–37. 10334035 10.1023/A:1008013117929 PMC7088537

[38] Xu W, Coombs KM (2008) Avian reovirus L2 genome segment sequences and predicted structure/function of the encoded RNA-dependent RNA polymerase protein. Virol J 5: 153. 19091125 10.1186/1743-422X-5-153 PMC2615760

[39] Bruenn JA (2003) A structural and primary sequence comparison of the viral RNA-dependent RNA polymerases. Nucleic Acids Res 31: 1821–1829. 12654997 10.1093/nar/gkg277 PMC152793

[40] O’Reilly EK, Kao CC (1998) Analysis of RNA-dependent RNA polymerase structure and function as guided by known polymerase structures and computer predictions of secondary structure. Virology 252: 287–303. 9878607 10.1006/viro.1998.9463

[41] Kamer G, Argos P (1984) Primary structural comparison of RNA-dependent polymerases from plant, animal and bacterial viruses. Nucleic Acids Res 12: 7269–7282. 6207485 10.1093/nar/12.18.7269 PMC320156

[42] Poch O, Sauvaget I, Delarue M, Tordo N (1989) Identification of four conserved motifs among the RNA-dependent polymerase encoding elements. EMBO J 8: 3867–3874. 2555175 10.1002/j.1460-2075.1989.tb08565.x PMC402075

[43] Morozov SY (1989) A possible relationship of reovirus putative RNA polymerase to polymerases of positive-strand RNA viruses. Nucleic Acids Res 17: 5394. 2548159 10.1093/nar/17.13.5394 PMC318133

[44] Hsiao J, Martinez-Costas J, Benavente J, Vakharia VN (2002) Cloning, expression, and characterization of avian reovirus guanylyltransferase. Virology 296: 288–299. 12069527 10.1006/viro.2002.1427

[45] Luongo CL, Contreras CM, Farsetta DL, Nibert ML (1998) Binding site for S-adenosyl-L-methionine in a central region of mammalian reovirus lambda2 protein. Evidence for activities in mRNA cap methylation. J Biol Chem 273: 23773–23780. 9726986 10.1074/jbc.273.37.23773

[46] Luongo CL, Reinisch KM, Harrison SC, Nibert ML (2000) Identification of the guanylyltransferase region and active site in reovirus mRNA capping protein lambda2. J Biol Chem 275: 2804–2810. 10644745 10.1074/jbc.275.4.2804

[47] Qiu T, Luongo CL (2003) Identification of two histidines necessary for reovirus mRNA guanylyltransferase activity. Virology 316: 313–324. 14644613 10.1016/j.virol.2003.08.027

[48] Buchan DW, Ward SM, Lobley AE, Nugent TC, Bryson K, et al (2010) Protein annotation and modelling servers at University College London. Nucleic Acids Res 38: W563–W568. 20507913 10.1093/nar/gkq427 PMC2896093

[49] Jones DT (1999) Protein secondary structure prediction based on position-specific scoring matrices. J Mol Biol 292: 195–202. 10493868 10.1006/jmbi.1999.3091

[50] Varela R, Benavente J (1994) Protein coding assignment of avian reovirus strain S1133. J Virol 68: 6775–6777. 8084013 10.1128/jvi.68.10.6775-6777.1994 PMC237102

[51] Bartlett JA, Joklik WK (1988) The sequence of the reovirus serotype 3 L3 genome segment which encodes the major core protein lambda 1. Virology 167: 31–37. 3267236 10.1016/0042-6822(88)90051-7

[52] Fang Q, Attoui H, Cantaloube JF, Biagini P, Zhu Z, et al (2000) Sequence of genome segments 1, 2, and 3 of the grass carp reovirus (Genus Aquareovirus, family Reoviridae). Biochem Biophys Res Commun 274: 762–766. 10924351 10.1006/bbrc.2000.3215

[53] Kim J, Tao Y, Reinisch KM, Harrison SC, Nibert ML (2004) Orthoreovirus and Aquareovirus core proteins: conserved enzymatic surfaces, but not protein-protein interfaces. Virus Res 101: 15–28. 15010214 10.1016/j.virusres.2003.12.003

[54] Reinisch KM, Nibert ML, Harrison SC (2000) Structure of the reovirus core at 3.6 A resolution. Nature 404: 960–967. 10801118 10.1038/35010041

[55] Bisaillon M, Lemay G (1997) Molecular dissection of the reovirus lambda1 protein nucleic acids binding site. Virus Res 51: 231–237. 9498620 10.1016/s0168-1702(97)00092-0

[56] Harrison SJ, Farsetta DL, Kim J, Noble S, Broering TJ, et al (1999) Mammalian reovirus L3 gene sequences and evidence for a distinct amino-terminal region of the lambda1 protein. Virology 258: 54–64. 10329567 10.1006/viro.1999.9707

[57] Lemay G, Danis C (1994) Reovirus lambda 1 protein: affinity for double-stranded nucleic acids by a small amino-terminal region of the protein independent from the zinc finger motif. J Gen Virol 75: 3261–3266. 7964637 10.1099/0022-1317-75-11-3261

[58] Xu W, Coombs KM (2009) Conserved structure/function of the orthoreovirus major core proteins. Virus Res 144: 44–57. 19720241 10.1016/j.virusres.2009.03.020 PMC5123878

[59] Wang L, Brown SJ (2006) BindN: a web-based tool for efficient prediction of DNA and RNA binding sites in amino acid sequences. Nucleic Acids Res 34: W243–W248. 16845003 10.1093/nar/gkl298 PMC1538853

[60] Coombs KM (1998) Stoichiometry of reovirus structural proteins in virus, ISVP, and core particles. Virology 243: 218–228. 9527931 10.1006/viro.1998.9061

[61] Brentano L, Noah DL, Brown EG, Sherry B (1998) The reovirus protein mu2, encoded by the M1 gene, is an RNA-binding protein. J Virol 72: 8354–8357. 9733883 10.1128/jvi.72.10.8354-8357.1998 PMC110211

[62] Noble S, Nibert ML (1997) Core protein mu2 is a second determinant of nucleoside triphosphatase activities by reovirus cores. J Virol 71: 7728–7735. 9311857 10.1128/jvi.71.10.7728-7735.1997 PMC192124

[63] Kim J, Parker JS, Murray KE, Nibert ML (2004) Nucleoside and RNA triphosphatase activities of orthoreovirus transcriptase cofactor mu2. J Biol Chem 279: 4394–4403. 14613938 10.1074/jbc.M308637200

[64] Su YP, Shien JH, Liu HJ, Yin HS, Lee LH (2007) Avian reovirus core protein muA expressed in Escherichia coli possesses both NTPase and RTPase activities. J Gen Virol 88: 1797–1805. 17485541 10.1099/vir.0.82592-0

[65] Kobayashi T, Ooms LS, Chappell JD, Dermody TS (2009) Identification of functional domains in reovirus replication proteins muNS and mu2. J Virol 83: 2892–2906. 19176625 10.1128/JVI.01495-08 PMC2655549

[66] Parker JS, Broering TJ, Kim J, Higgins DE, Nibert ML (2002) Reovirus core protein mu2 determines the filamentous morphology of viral inclusion bodies by interacting with and stabilizing microtubules. J Virol 76: 4483–4496. 11932414 10.1128/JVI.76.9.4483-4496.2002 PMC155082

[67] Yin P, Keirstead ND, Broering TJ, Arnold MM, Parker JS, et al (2004) Comparisons of the M1 genome segments and encoded mu2 proteins of different reovirus isolates. Virol J 1: 6. 15507160 10.1186/1743-422X-1-6 PMC524354

[68] Touris-Otero F, Cortez-San MM, Martinez-Costas J, Benavente J (2004) Avian reovirus morphogenesis occurs within viral factories and begins with the selective recruitment of sigmaNS and lambdaA to microNS inclusions. J Mol Biol 341: 361–374. 15276829 10.1016/j.jmb.2004.06.026

[69] Irvin SC, Zurney J, Ooms LS, Chappell JD, Dermody TS, et al (2012) A single-amino-acid polymorphism in reovirus protein mu2 determines repression of interferon signaling and modulates myocarditis. J Virol 86: 2302–2311. 22156521 10.1128/JVI.06236-11 PMC3302381

[70] Jayasuriya AK, Nibert ML, Fields BN (1988) Complete nucleotide sequence of the M2 gene segment of reovirus type 3 dearing and analysis of its protein product mu 1. Virology 163: 591–602. 3354207 10.1016/0042-6822(88)90300-5

[71] Wiener JR, Joklik WK (1988) Evolution of reovirus genes: a comparison of serotype 1, 2, and 3 M2 genome segments, which encode the major structural capsid protein mu 1C. Virology 163: 603–613. 3354208 10.1016/0042-6822(88)90301-7

[72] Tillotson L, Shatkin AJ (1992) Reovirus polypeptide sigma 3 and N-terminal myristoylation of polypeptide mu 1 are required for site-specific cleavage to mu 1C in transfected cells. J Virol 66: 2180–2186. 1548757 10.1128/jvi.66.4.2180-2186.1992 PMC289010

[73] Varela R, Martinez-Costas J, Mallo M, Benavente J (1996) Intracellular posttranslational modifications of S1133 avian reovirus proteins. J Virol 70: 2974–2981. 8627773 10.1128/jvi.70.5.2974-2981.1996 PMC190156

[74] Odegard AL, Chandran K, Zhang X, Parker JS, Baker TS, et al (2004) Putative autocleavage of outer capsid protein micro1, allowing release of myristoylated peptide micro1N during particle uncoating, is critical for cell entry by reovirus. J Virol 78: 8732–8745. 15280481 10.1128/JVI.78.16.8732-8745.2004 PMC479062

[75] Nibert ML, Odegard AL, Agosto MA, Chandran K, Schiff LA (2005) Putative autocleavage of reovirus mu1 protein in concert with outer-capsid disassembly and activation for membrane permeabilization. J Mol Biol 345: 461–474. 15581891 10.1016/j.jmb.2004.10.026

[76] McCrae MA, Joklik WK (1978) The nature of the polypeptide encoded by each of the 10 double-stranded RNA segments of reovirus type 3. Virology 89: 578–593. 716218 10.1016/0042-6822(78)90199-x

[77] Mustoe TA, Ramig RF, Sharpe AH, Fields BN (1978) Genetics of reovirus: identification of the ds RNA segments encoding the polypeptides of the mu and sigma size classes. Virology 89: 594–604. 716219 10.1016/0042-6822(78)90200-3

[78] Noad L, Shou J, Coombs KM, Duncan R (2006) Sequences of avian reovirus M1, M2 and M3 genes and predicted structure/function of the encoded mu proteins. Virus Res 116: 45–57. 16297481 10.1016/j.virusres.2005.08.014 PMC5123877

[79] Ivanovic T, Boulant S, Ehrlich M, Demidenko AA, Arnold MM, et al (2011) Recruitment of cellular clathrin to viral factories and disruption of clathrin-dependent trafficking. Traffic 12: 1179–1195. 21736684 10.1111/j.1600-0854.2011.01233.x PMC3575638

[80] Mora M, Partin K, Bhatia M, Partin J, Carter C (1987) Association of reovirus proteins with the structural matrix of infected cells. Virology 159: 265–277. 3617500 10.1016/0042-6822(87)90464-8

[81] Broering TJ, Kim J, Miller CL, Piggott CD, Dinoso JB, et al (2004) Reovirus nonstructural protein mu NS recruits viral core surface proteins and entering core particles to factory-like inclusions. J Virol 78: 1882–1892. 14747553 10.1128/JVI.78.4.1882-1892.2004 PMC369481

[82] Broering TJ, McCutcheon AM, Centonze VE, Nibert ML (2000) Reovirus nonstructural protein muNS binds to core particles but does not inhibit their transcription and capping activities. J Virol 74: 5516–5524. 10823857 10.1128/jvi.74.12.5516-5524.2000 PMC112037

[83] Broering TJ, Parker JS, Joyce PL, Kim J, Nibert ML (2002) Mammalian reovirus nonstructural protein microNS forms large inclusions and colocalizes with reovirus microtubule-associated protein micro2 in transfected cells. J Virol 76: 8285–8297. 12134034 10.1128/JVI.76.16.8285-8297.2002 PMC155143

[84] Miller CL, Arnold MM, Broering TJ, Hastings CE, Nibert ML (2010) Localization of mammalian orthoreovirus proteins to cytoplasmic factory-like structures via nonoverlapping regions of microNS. J Virol 84: 867–882. 19889754 10.1128/JVI.01571-09 PMC2798337

[85] Miller CL, Broering TJ, Parker JS, Arnold MM, Nibert ML (2003) Reovirus sigma NS protein localizes to inclusions through an association requiring the mu NS amino terminus. J Virol 77: 4566–4576. 12663763 10.1128/JVI.77.8.4566-4576.2003 PMC152138

[86] Brandariz-Nunez A, Menaya-Vargas R, Benavente J, Martinez-Costas J (2010) Avian reovirus microNS protein forms homo-oligomeric inclusions in a microtubule-independent fashion, which involves specific regions of its C-terminal domain. J Virol 84: 4289–4301. 20181708 10.1128/JVI.02534-09 PMC2863718

[87] Touris-Otero F, Martinez-Costas J, Vakharia VN, Benavente J (2004) Avian reovirus nonstructural protein microNS forms viroplasm-like inclusions and recruits protein sigmaNS to these structures. Virology 319: 94–106. 14967491 10.1016/j.virol.2003.10.034

[88] McCutcheon AM, Broering TJ, Nibert ML (1999) Mammalian reovirus M3 gene sequences and conservation of coiled-coil motifs near the carboxyl terminus of the microNS protein. Virology 264: 16–24. 10544126 10.1006/viro.1999.9990

[89] Cai L, Sun X, Shao L, Fang Q (2011) Functional investigation of grass carp reovirus nonstructural protein NS80. Virol J 8: 168. 21489306 10.1186/1743-422X-8-168 PMC3101161

[90] Broering TJ, Arnold MM, Miller CL, Hurt JA, Joyce PL, et al (2005) Carboxyl-proximal regions of reovirus nonstructural protein muNS necessary and sufficient for forming factory-like inclusions. J Virol 79: 6194–6206. 15858004 10.1128/JVI.79.10.6194-6206.2005 PMC1091696

[91] Wiener JR, Bartlett JA, Joklik WK (1989) The sequences of reovirus serotype 3 genome segments M1 and M3 encoding the minor protein mu 2 and the major nonstructural protein mu NS, respectively. Virology 169: 293–304. 2523177 10.1016/0042-6822(89)90154-2

[92] Busch LK, Rodriguez-Grille J, Casal JI, Martinez-Costas J, Benavente J (2011) Avian and mammalian reoviruses use different molecular mechanisms to synthesize their {micro}NS isoforms. J Gen Virol 92: 2566–2574. 21795469 10.1099/vir.0.036459-0

[93] Kozak M (1991) An analysis of vertebrate mRNA sequences: intimations of translational control. J Cell Biol 115: 887–903. 1955461 10.1083/jcb.115.4.887 PMC2289952

[94] Schnitzer TJ, Ramos T, Gouvea V (1982) Avian reovirus polypeptides: analysis of intracellular virus-specified products, virions, top component, and cores. J Virol 43: 1006–1014. 7143561 10.1128/jvi.43.3.1006-1014.1982 PMC256211

[95] Yin HS, Shieh HK, Lee LH (1997) Characterization of the double-stranded RNA genome segment S3 of avian reovirus. J Virol Methods 67: 93–101. 9274822 10.1016/s0166-0934(97)00080-3

[96] Key T, Read J, Nibert ML, Duncan R (2013) Piscine Reovirus Encodes a Cytotoxic, Nonfusogenic, Integral Membrane Protein and Previously Unrecognized Virion Outer-Capsid Proteins. J Gen Virol 94: 1039–50. 23343626 10.1099/vir.0.048637-0

[97] Schiff LA, Nibert ML, Co MS, Brown EG, Fields BN (1988) Distinct binding sites for zinc and double-stranded RNA in the reovirus outer capsid protein sigma 3. Mol Cell Biol 8: 273–283. 3275869 10.1128/mcb.8.1.273-283.1988 PMC363116

[98] Martinez-Costas J, Grande A, Varela R, Garcia-Martinez C, Benavente J (1997) Protein architecture of avian reovirus S1133 and identification of the cell attachment protein. J Virol 71: 59–64. 8985323 10.1128/jvi.71.1.59-64.1997 PMC191024

[99] Cheng L, Fang Q, Shah S, Atanasov IC, Zhou ZH (2008) Subnanometer-resolution structures of the grass carp reovirus core and virion. J Mol Biol 382: 213–222. 18625243 10.1016/j.jmb.2008.06.075 PMC2900196

[100] Guardado-Calvo P, Vazquez-Iglesias L, Martinez-Costas J, Llamas-Saiz AL, Schoehn G, et al (2008) Crystal structure of the avian reovirus inner capsid protein sigmaA. J Virol 82: 11208–11216. 18799570 10.1128/JVI.00733-08 PMC2573251

[101] Yin HS, Shien JH, Lee LH (2000) Synthesis in Escherichia coli of avian reovirus core protein varsigmaA and its dsRNA-binding activity. Virology 266: 33–41. 10612658 10.1006/viro.1999.0020

[102] Dermody TS, Schiff LA, Nibert ML, Coombs KM, Fields BN (1991) The S2 gene nucleotide sequences of prototype strains of the three reovirus serotypes: characterization of reovirus core protein sigma 2. J Virol 65: 5721–5731. 1920614 10.1128/jvi.65.11.5721-5731.1991 PMC250232

[103] Richardson MA, Furuichi Y (1983) Nucleotide sequence of reovirus genome segment S3, encoding non-structural protein sigma NS. Nucleic Acids Res 11: 6399–6408. 6312421 10.1093/nar/11.18.6399 PMC326381

[104] Gillian AL, Nibert ML (1998) Amino terminus of reovirus nonstructural protein sigma NS is important for ssRNA binding and nucleoprotein complex formation. Virology 240: 1–11. 9448684 10.1006/viro.1997.8905

[105] la Cour T, Kiemer L, Molgaard A, Gupta R, Skriver K, et al (2004) Analysis and prediction of leucine-rich nuclear export signals. Protein Eng Des Sel 17: 527–536. 15314210 10.1093/protein/gzh062

[106] Weiner HL, Ault KA, Fields BN (1980) Interaction of reovirus with cell surface receptors. I. Murine and human lymphocytes have a receptor for the hemagglutinin of reovirus type 3. J Immunol 124: 2143–2148. 7365250

[107] Ernst H, Shatkin AJ (1985) Reovirus hemagglutinin mRNA codes for two polypeptides in overlapping reading frames. Proc Natl Acad Sci U S A 82: 48–52. 3855548 10.1073/pnas.82.1.48 PMC396968

[108] Bodelon G, Labrada L, Martinez-Costas J, Benavente J (2001) The avian reovirus genome segment S1 is a functionally tricistronic gene that expresses one structural and two nonstructural proteins in infected cells. Virology 290: 181–191. 11883183 10.1006/viro.2001.1159

[109] Grande A, Costas C, Benavente J (2002) Subunit composition and conformational stability of the oligomeric form of the avian reovirus cell-attachment protein sigmaC. J Gen Virol 83: 131–139. 11752709 10.1099/0022-1317-83-1-131

[110] Shaw AL, Samal SK, Subramanian K, Prasad BV (1996) The structure of aquareovirus shows how the different geometries of the two layers of the capsid are reconciled to provide symmetrical interactions and stabilization. Structure 4: 957–967. 8805574 10.1016/s0969-2126(96)00102-5

[111] Fraser RD, Furlong DB, Trus BL, Nibert ML, Fields BN, et al (1990) Molecular structure of the cell-attachment protein of reovirus: correlation of computer-processed electron micrographs with sequence-based predictions. J Virol 64: 2990–3000. 2335824 10.1128/jvi.64.6.2990-3000.1990 PMC249483

[112] Nibert ML, Dermody TS, Fields BN (1990) Structure of the reovirus cell-attachment protein: a model for the domain organization of sigma 1. J Virol 64: 2976–2989. 2335823 10.1128/jvi.64.6.2976-2989.1990 PMC249482

[113] Shapouri MR, Kane M, Letarte M, Bergeron J, Arella M, et al (1995) Cloning, sequencing and expression of the S1 gene of avian reovirus. J Gen Virol 76: 1515–1520. 7782781 10.1099/0022-1317-76-6-1515

[114] Strong JE, Leone G, Duncan R, Sharma RK, Lee PW (1991) Biochemical and biophysical characterization of the reovirus cell attachment protein sigma 1: evidence that it is a homotrimer. Virology 184: 23–32. 1871968 10.1016/0042-6822(91)90818-V PMC7130766

[115] Barton ES, Forrest JC, Connolly JL, Chappell JD, Liu Y, et al (2001) Junction adhesion molecule is a receptor for reovirus. Cell 104: 441–451. 11239401 10.1016/s0092-8674(01)00231-8

[116] Dryden KA, Wang G, Yeager M, Nibert ML, Coombs KM, et al (1993) Early steps in reovirus infection are associated with dramatic changes in supramolecular structure and protein conformation: analysis of virions and subviral particles by cryoelectron microscopy and image reconstruction. J Cell Biol 122: 1023–1041. 8394844 10.1083/jcb.122.5.1023 PMC2119633

[117] Chappell JD, Duong JL, Wright BW, Dermody TS (2000) Identification of carbohydrate-binding domains in the attachment proteins of type 1 and type 3 reoviruses. J Virol 74: 8472–8479. 10954547 10.1128/jvi.74.18.8472-8479.2000 PMC116358

[118] Chappell JD, Prota AE, Dermody TS, Stehle T (2002) Crystal structure of reovirus attachment protein sigma1 reveals evolutionary relationship to adenovirus fiber. EMBO J 21: 1–11. 11782420 10.1093/emboj/21.1.1 PMC125343

[119] Campbell JA, Schelling P, Wetzel JD, Johnson EM, Forrest JC, et al (2005) Junctional adhesion molecule a serves as a receptor for prototype and field-isolate strains of mammalian reovirus. J Virol 79: 7967–7978. 15956543 10.1128/JVI.79.13.7967-7978.2005 PMC1143703

[120] Gentsch JR, Pacitti AF (1985) Effect of neuraminidase treatment of cells and effect of soluble glycoproteins on type 3 reovirus attachment to murine L cells. J Virol 56: 356–364. 4057353 10.1128/jvi.56.2.356-364.1985 PMC252582

[121] Paul RW, Choi AH, Lee PW (1989) The alpha-anomeric form of sialic acid is the minimal receptor determinant recognized by reovirus. Virology 172: 382–385. 2773327 10.1016/0042-6822(89)90146-3

[122] Dermody TS, Nibert ML, Bassel-Duby R, Fields BN (1990) A sigma 1 region important for hemagglutination by serotype 3 reovirus strains. J Virol 64: 5173–5176. 2398540 10.1128/jvi.64.10.5173-5176.1990 PMC248012

[123] Chappell JD, Gunn VL, Wetzel JD, Baer GS, Dermody TS (1997) Mutations in type 3 reovirus that determine binding to sialic acid are contained in the fibrous tail domain of viral attachment protein sigma1. J Virol 71: 1834–1841. 9032313 10.1128/jvi.71.3.1834-1841.1997 PMC191253

[124] Reiter DM, Frierson JM, Halvorson EE, Kobayashi T, Dermody TS, et al (2011) Crystal structure of reovirus attachment protein sigma1 in complex with sialylated oligosaccharides. PLoS Pathog 7: e1002166. 21829363 10.1371/journal.ppat.1002166 PMC3150272

[125] Racine T, Hurst T, Barry C, Shou J, Kibenge F, et al (2009) Aquareovirus effects syncytiogenesis by using a novel member of the FAST protein family translated from a noncanonical translation start site. J Virol 83: 5951–5955. 19297495 10.1128/JVI.00171-09 PMC2681948

[126] Guo H, Sun X, Yan L, Shao L, Fang Q (2013) The NS16 protein of aquareovirus-C is a fusion-associated small transmembrane (FAST) protein, and its activity can be enhanced by the nonstructural protein NS26. Virus Res 171: 129–137. 23201583 10.1016/j.virusres.2012.11.011

[127] Shmulevitz M, Duncan R (2000) A new class of fusion-associated small transmembrane (FAST) proteins encoded by the non-enveloped fusogenic reoviruses. EMBO J 19: 902–912. 10698932 10.1093/emboj/19.5.902 PMC305630

[128] Ke F, He LB, Pei C, Zhang QY (2011) Turbot reovirus (SMReV) genome encoding a FAST protein with a non-AUG start site. BMC Genomics 12: 323. 21689389 10.1186/1471-2164-12-323 PMC3135578

[129] Dawe S, Duncan R (2002) The S4 genome segment of baboon reovirus is bicistronic and encodes a novel fusion-associated small transmembrane protein. J Virol 76: 2131–2140. 11836390 10.1128/jvi.76.5.2131-2140.2002 PMC135948

[130] Corcoran JA, Duncan R (2004) Reptilian reovirus utilizes a small type III protein with an external myristylated amino terminus to mediate cell-cell fusion. J Virol 78: 4342–4351. 15047847 10.1128/JVI.78.8.4342-4351.2004 PMC374291

[131] Boutilier J, Duncan R (2011) The reovirus fusion-associated small transmembrane (FAST) proteins: virus-encoded cellular fusogens. Curr Top Membr 68: 107–140. 21771497 10.1016/B978-0-12-385891-7.00005-2

[132] Touris-Otero F, Martinez-Costas J, Vakharia VN, Benavente J (2005) Characterization of the nucleic acid-binding activity of the avian reovirus non-structural protein sigma NS. J Gen Virol 86: 1159–1169. 15784910 10.1099/vir.0.80491-0

[133] Zurney J, Kobayashi T, Holm GH, Dermody TS, Sherry B (2009) Reovirus mu2 protein inhibits interferon signaling through a novel mechanism involving nuclear accumulation of interferon regulatory factor 9. J Virol 83: 2178–2187. 19109390 10.1128/JVI.01787-08 PMC2643726

[134] Gonzalez-Lopez C, Martinez-Costas J, Esteban M, Benavente J (2003) Evidence that avian reovirus sigmaA protein is an inhibitor of the double-stranded RNA-dependent protein kinase. J Gen Virol 84: 1629–1639. 12771434 10.1099/vir.0.19004-0

[135] Martinez-Costas J, Gonzalez-Lopez C, Vakharia VN, Benavente J (2000) Possible involvement of the double-stranded RNA-binding core protein sigmaA in the resistance of avian reovirus to interferon. J Virol 74: 1124–1131. 10627522 10.1128/jvi.74.3.1124-1131.2000 PMC111446

[136] Sherry B (2009) Rotavirus and reovirus modulation of the interferon response. J Interferon Cytokine Res 29: 559–567. 19694545 10.1089/jir.2009.0072 PMC2956631

[137] Imani F, Jacobs BL (1988) Inhibitory activity for the interferon-induced protein kinase is associated with the reovirus serotype 1 sigma 3 protein. Proc Natl Acad Sci U S A 85: 7887–7891. 2460857 10.1073/pnas.85.21.7887 PMC282302

[138] Lauksund S, Svingerud T, Bergan V, Robertsen B (2009) Atlantic salmon IPS-1 mediates induction of IFNa1 and activation of NF-kappaB and localizes to mitochondria. Dev Comp Immunol 33: 1196–1204. 19576240 10.1016/j.dci.2009.06.012

[139] Bergan V, Steinsvik S, Xu H, Kileng Ø, Robertsen B (2006) Promoters of type I interferon genes from Atlantic salmon contain two main regulatory regions. FEBS J 273: 3893–3906. 16889635 10.1111/j.1742-4658.2006.05382.x

[140] Boehme KW, Frierson JM, Konopka JL, Kobayashi T, Dermody TS (2011) The reovirus sigma1s protein is a determinant of hematogenous but not neural virus dissemination in mice. J Virol 85: 11781–11790. 21917967 10.1128/JVI.02289-10 PMC3209282

[141] Boehme KW, Guglielmi KM, Dermody TS (2009) Reovirus nonstructural protein sigma1s is required for establishment of viremia and systemic dissemination. Proc Natl Acad Sci U S A 106: 19986–19991. 19897716 10.1073/pnas.0907412106 PMC2774258

[142] Hoyt CC, Richardson-Burns SM, Goody RJ, Robinson BA, Debiasi RL, et al (2005) Nonstructural protein sigma1s is a determinant of reovirus virulence and influences the kinetics and severity of apoptosis induction in the heart and central nervous system. J Virol 79: 2743–2753. 15708993 10.1128/JVI.79.5.2743-2753.2005 PMC548430

[143] Poggioli GJ, Keefer C, Connolly JL, Dermody TS, Tyler KL (2000) Reovirus-induced G(2)/M cell cycle arrest requires sigma1s and occurs in the absence of apoptosis. J Virol 74: 9562–9570. 11000227 10.1128/jvi.74.20.9562-9570.2000 PMC112387

[144] Chulu JL, Huang WR, Wang L, Shih WL, Liu HJ (2010) Avian reovirus nonstructural protein p17-induced G(2)/M cell cycle arrest and host cellular protein translation shutoff involve activation of p53-dependent pathways. J Virol 84: 7683–7694. 20484520 10.1128/JVI.02604-09 PMC2897625

[145] Costas C, Martinez-Costas J, Bodelon G, Benavente J (2005) The second open reading frame of the avian reovirus S1 gene encodes a transcription-dependent and CRM1-independent nucleocytoplasmic shuttling protein. J Virol 79: 2141–2150. 15681417 10.1128/JVI.79.4.2141-2150.2005 PMC546569

[146] John KR, George MR, Richards RH, Frerichs GN (2001) Characteristics of a new reovirus isolated from epizootic ulcerative syndrome infected snakehead fish. Dis Aquat Organ 46: 83–92. 11678232 10.3354/dao046083

[147] Wolf E, Kim PS, Berger B (1997) MultiCoil: a program for predicting two- and three-stranded coiled coils. Protein Sci 6: 1179–1189. 9194178 10.1002/pro.5560060606 PMC2143730

[148] Lupas A, Van DM, Stock J (1991) Predicting coiled coils from protein sequences. Science 252: 1162–1164. 2031185 10.1126/science.252.5009.1162

[149] Kyte J, Doolittle RF (1982) A simple method for displaying the hydropathic character of a protein. J Mol Biol 157: 105–132. 7108955 10.1016/0022-2836(82)90515-0

[150] Ren J, Wen L, Gao X, Jin C, Xue Y, et al (2008) CSS-Palm 2.0: an updated software for palmitoylation sites prediction. Protein Eng Des Sel 21: 639–644. 18753194 10.1093/protein/gzn039 PMC2569006

