# Correction: Sequence Analysis of the Genome of Piscine Orthoreovirus (PRV) Associated with Heart and Skeletal Muscle Inflammation (HSMI) in Atlantic Salmon (*Salmo salar*)

**DOI:** 10.1371/annotation/746a9036-0e54-4a9a-ab16-adb108a3a227

**Published:** 2013-08-02

**Authors:** Turhan Markussen, Maria K. Dahle, Torstein Tengs, Marie Løvoll, Øystein W. Finstad, Christer R. Wiik-Nielsen, Søren Grove, Silje Lauksund, Børre Robertsen, Espen Rimstad

Figures 2 and 3 were accidentally switched. The image appearing as Figure 2 belongs with the title and legend for Figure 3, and the image appearing as Figure 3 belongs with the title and legend for Figure 2. The figure titles and legends themselves are in correct order. 

