# Supplementary figures and images for: Sequence Analysis of the Genome of Piscine Orthoreovirus (PRV) Associated with Heart and Skeletal Muscle Inflammation (HSMI) in Atlantic Salmon (Salmo salar)
Source: PLoS One. 2013 Jul 29;8(7):e70075. doi: 10.1371/journal.pone.0070075 (PMC3726481; doi:10.1371/journal.pone.0070075)

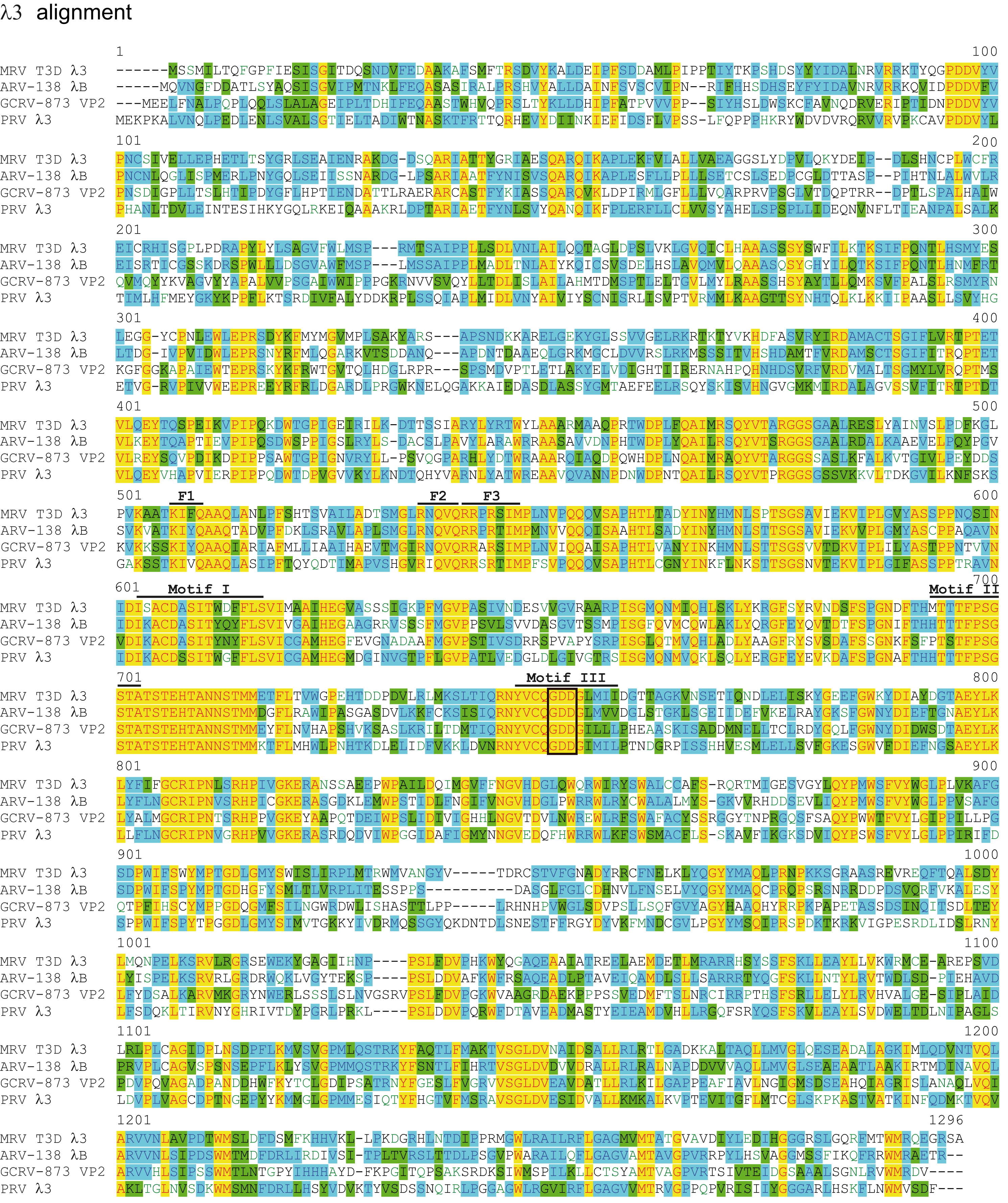

Supplement: Figure S1 — Multiple sequence alignment of PRV L1 ORF (λ3) with corresponding ORFs from the reovirus prototype strains MRV T3D, ARV-138 and GCRV-873. RNA-dependent RNA polymerase (RdRp) domains are indicated with the universally conserved GDD domain (in Motif III) boxed. (TIF) [file pone.0070075.s001.tif]

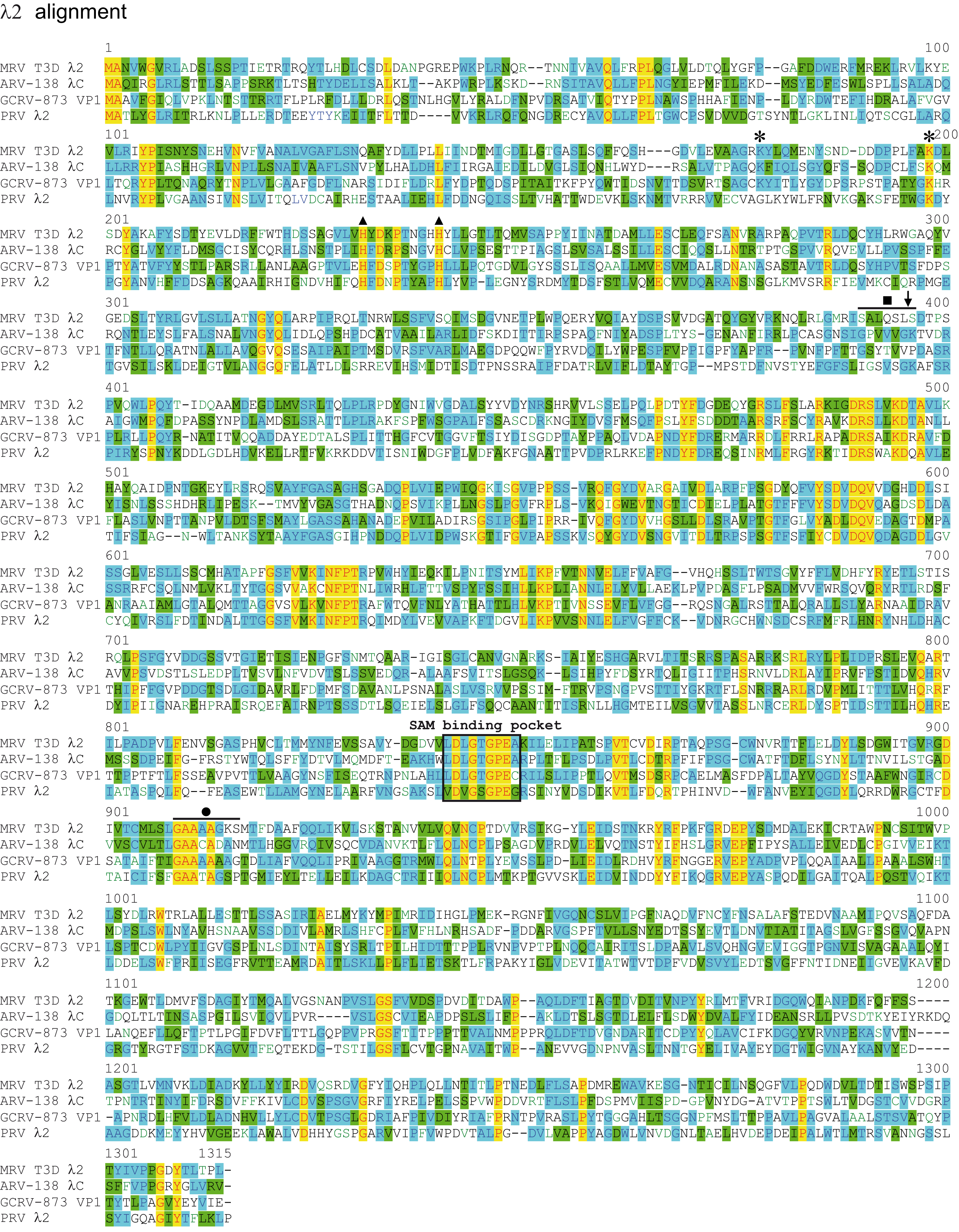

Supplement: Figure S2 — Multiple sequence alignment of PRV L2 ORF (λ2) with guanylyltransferases from the reovirus prototype strains MRV T3D, ARV-138 and GCRV-873. * = lysine residues in MRV essential (K190) or significant contributor (K171) for autoguanylation in the MRV, ARV and GCRV proteins, ▴ = conserved histidines essential for guanylyltransferase activity in the MRV protein, ▪ = ATP/GTP binding site motif A in ARV, boxed = S-adenosyl-L-methionine (SAM) binding pocket, • = ATP/GTP binding site motif A in MRV, and ↓ = hypersensitive cleavage site in recombinant MRV λ2 and ARV 1733 λC. (TIF) [file pone.0070075.s002.tif]

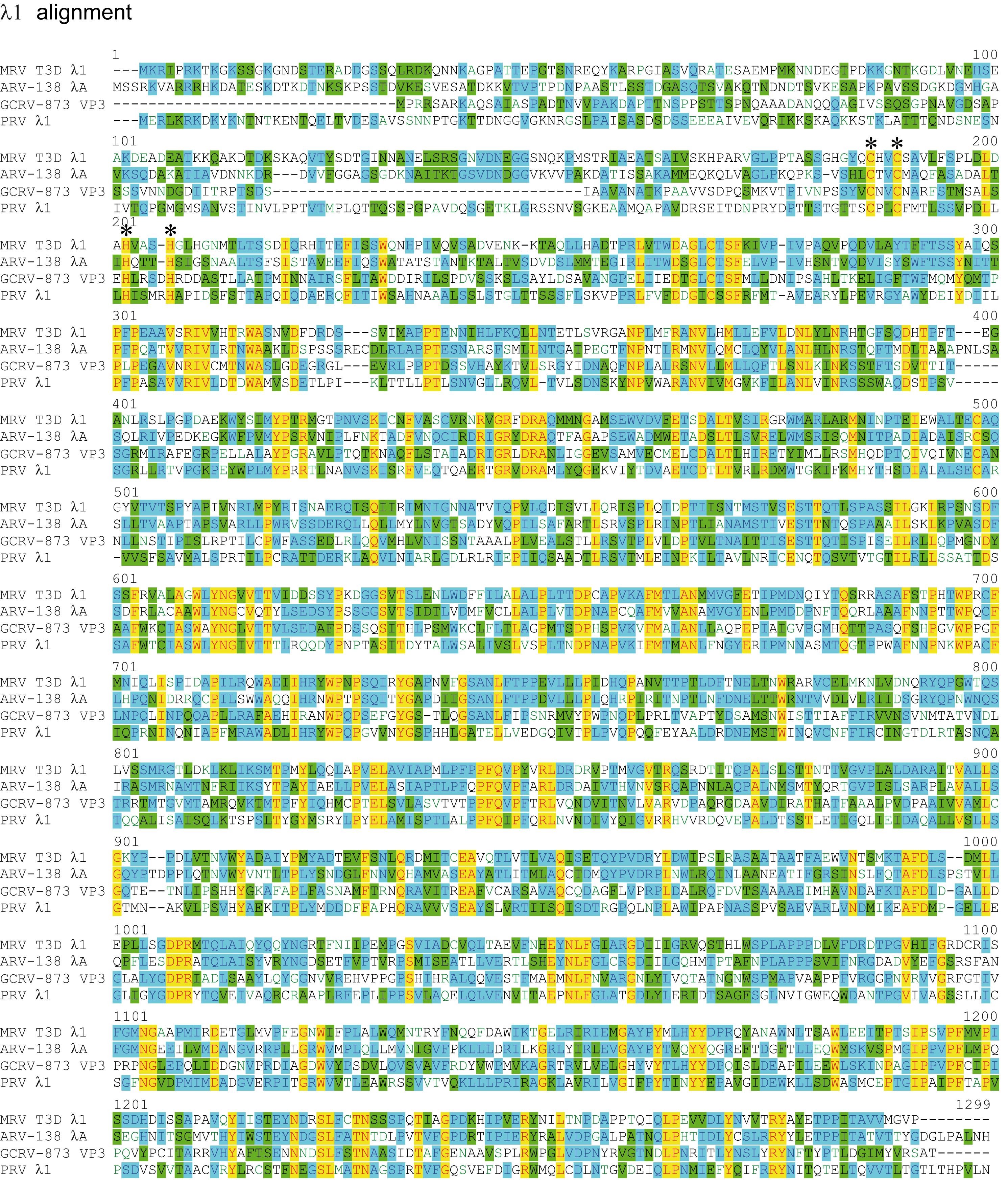

Supplement: Figure S3 — Multiple sequence alignment of PRV L3 ORF (λ1) with the helicase-NTPase/core capsid shell proteins from the reovirus prototype strains MRV T3D, ARV-138 and GCRV-873. * = conserved CCHH zinc-finger motif. (TIF) [file pone.0070075.s003.tif]

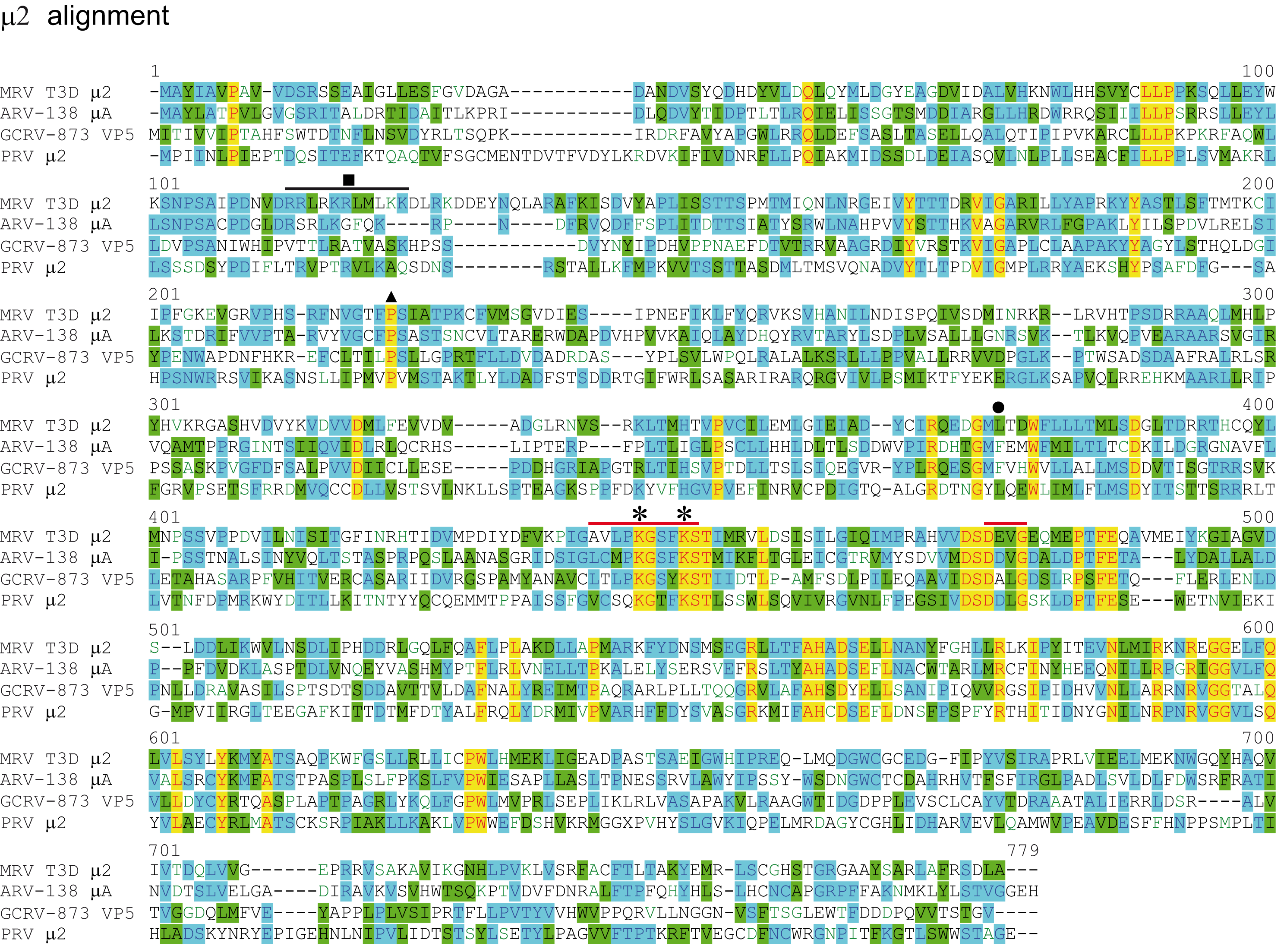

Supplement: Figure S4 — Multiple sequence alignment of PRV M1 ORF encoding the μ2 protein with the homologues proteins from the reovirus prototype strains MRV T3D, ARV-138 and GCRV-873. ▴ = conserved proline residue suggested to play a key role in the formation and structural organisation of reovirus inclusion bodies, a determinant of type I IFN antagonism and a modulator of myocarditis in neonatal mice. • = leucine vs. phenylananine, a determinant of tissue tropism of MRV μ2 in MDCK cells. ▪ = possible NLS in MRV. Red lines = nucleotide binding/triphosphate phosphohydrolase regions, and * = conserved lysine residues essential for ATPase activity in ARV µA. A nuclear export signal (NES) has been predicted for MRV μ2 (residues 328–335) [151]. NetNES 1.1 predicts a NES in ARV µA in the same region, while in GCRV, L233, L238 and L607 are predicted to participate in a NES, and in PRV L80 (numbering according to the GCRV and PRV sequences, respectively) (not shown). (TIF) [file pone.0070075.s004.tif]

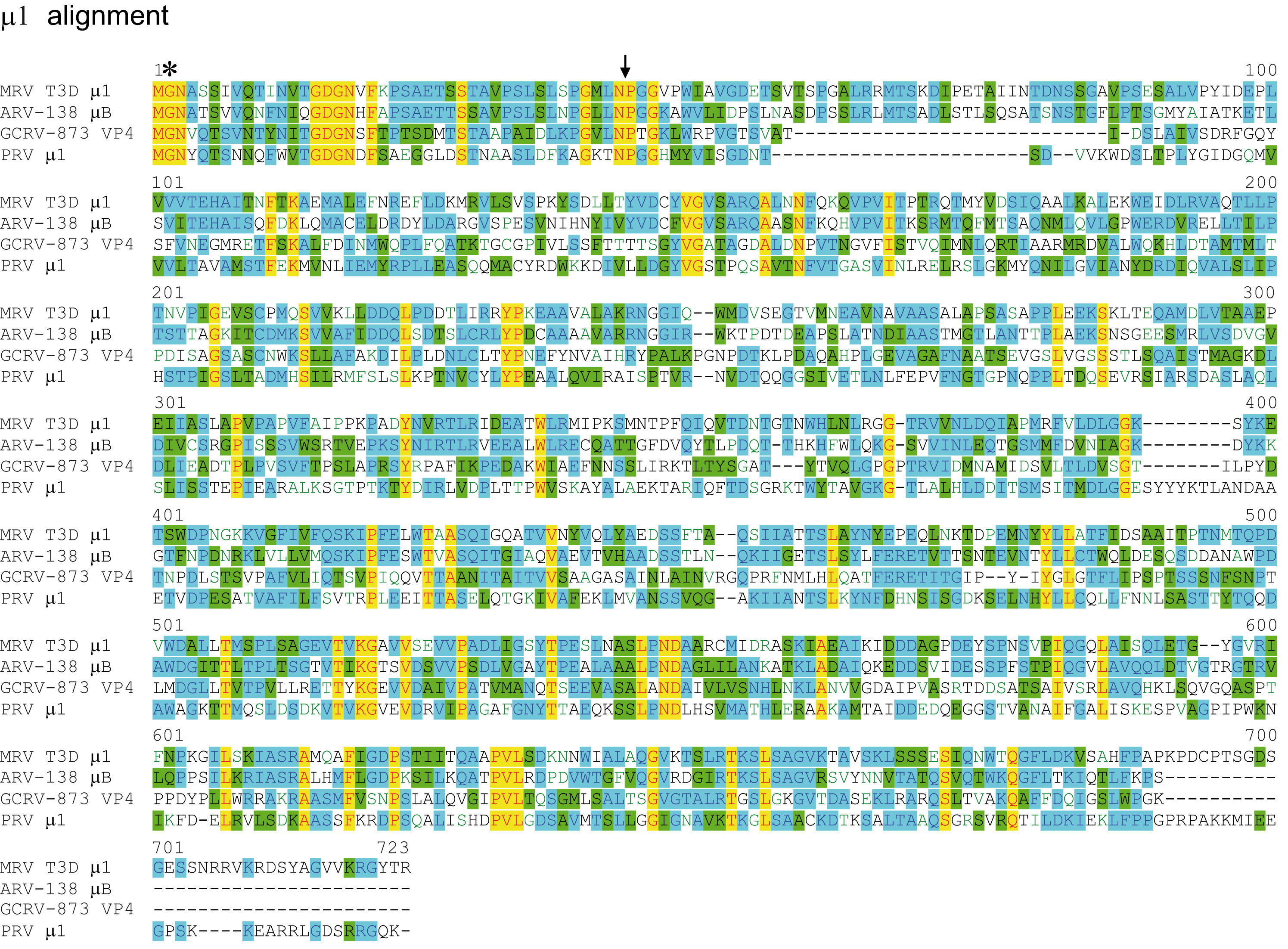

Supplement: Figure S5 — Multiple sequence alignment of PRV M2 ORF encoding the μ1/µB major outer capsid protein with the homologues proteins from the reovirus prototype strains MRV T3D, ARV-138 and GCRV-873. * = myristoylation site in the MRV protein. ↓ = post-translational cleavage site producing N- and C-terminal fragment μ1N and μ1C (MRV) or µBN and µBC (ARV). The C-terminal end of the MRV protein is extended by 33 amino acids compared to the homologous proteins in ARV and GCRV. The PRV protein is also extended, by 28 amino acids. (TIF) [file pone.0070075.s005.tif]

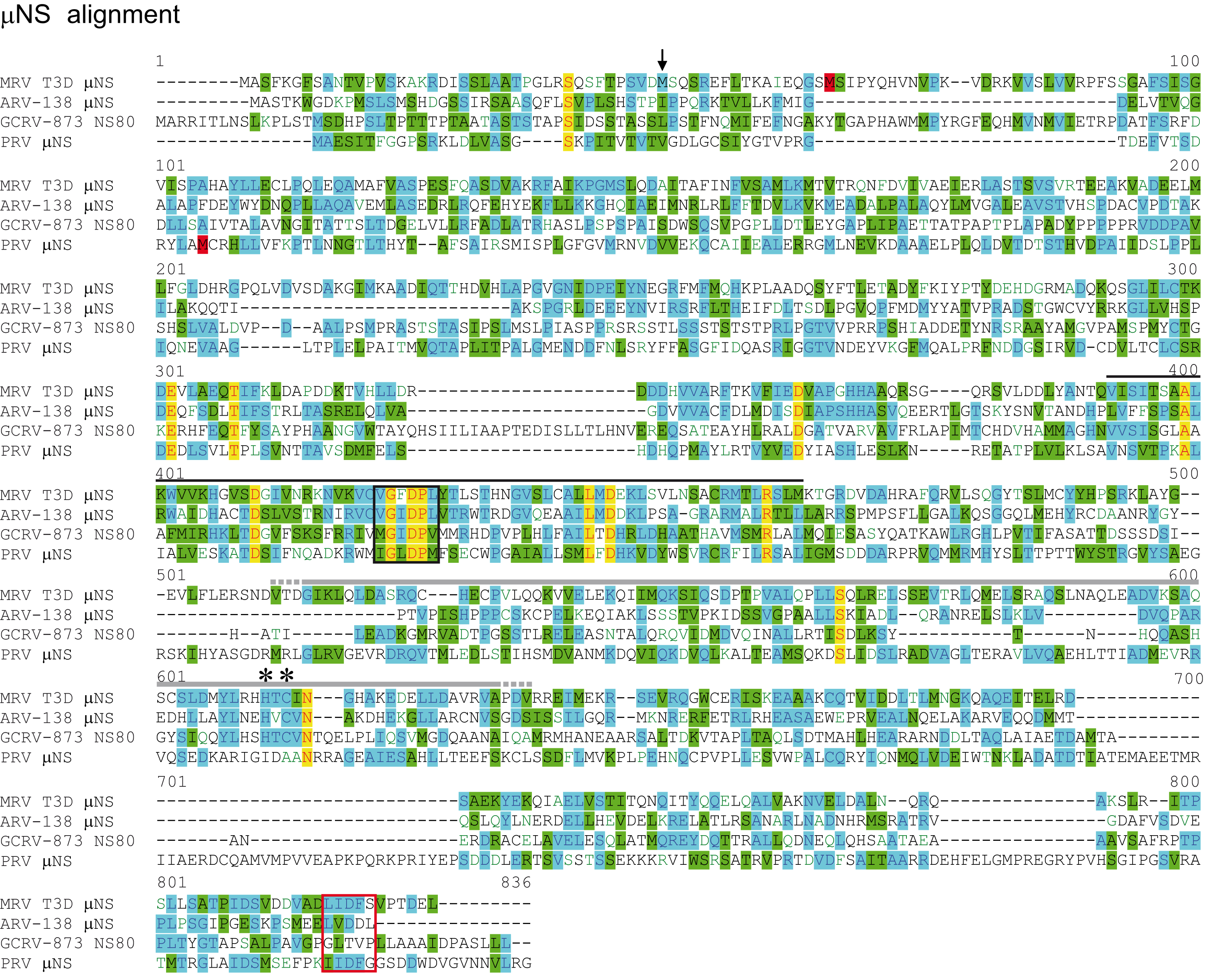

Supplement: Figure S6 — Multiple sequence alignment of PRV M3 ORF encoding the putative µNS protein aligned with µNS/NS80 proteins from the reovirus prototype strains MRV T3D, ARV-138 and GCRV-873. ↓ = N-terminal end of second translation product of MRV (µNSC). Met-57, conserved in MRV and PRV is boxed red. * = conserved putative zinc-hook motif crucial in the formation of inclusion-like structures in the MRV protein [86,90,152]. Black lines indicate sequence regions with higher level of conservation with the motif XGXDPX being boxed. In ARV, the larger region forms part of a region that has been shown to be involved in inclusion maturation [86]. Grey solid and dotted lines = coil-coil(s) regions as predicted by MultiCoil (window size: 21, probability cutoff: 0,5). The MRV L711IDFS715 motif shown to be required for the recruitment of clathrin to viral factories is boxed red. (TIF) [file pone.0070075.s006.tif]

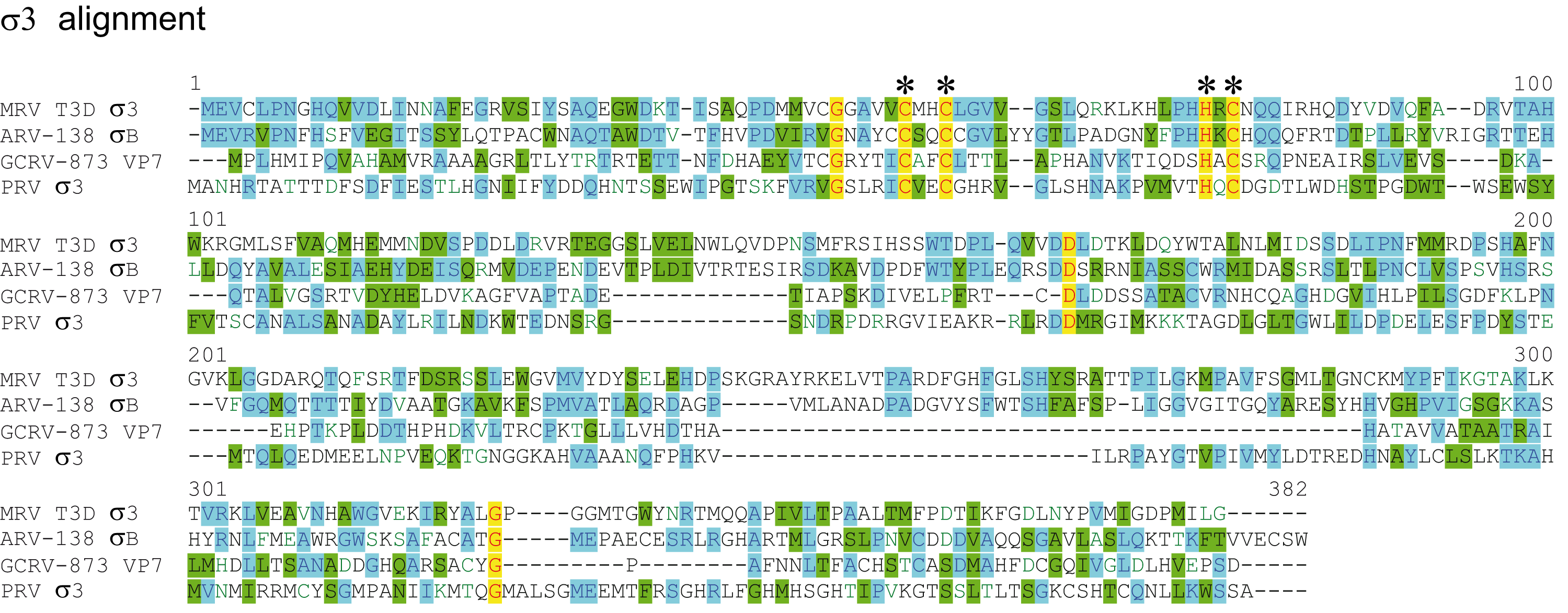

Supplement: Figure S7 — Multiple sequence alignment of PRV S1 ORF encoding the major outer capsid σ3 protein with the homologues proteins from the reovirus prototype strains MRV T3D, ARV-138 and GCRV-873. * = conserved Zn-finger motif. (TIF) [file pone.0070075.s007.tif]

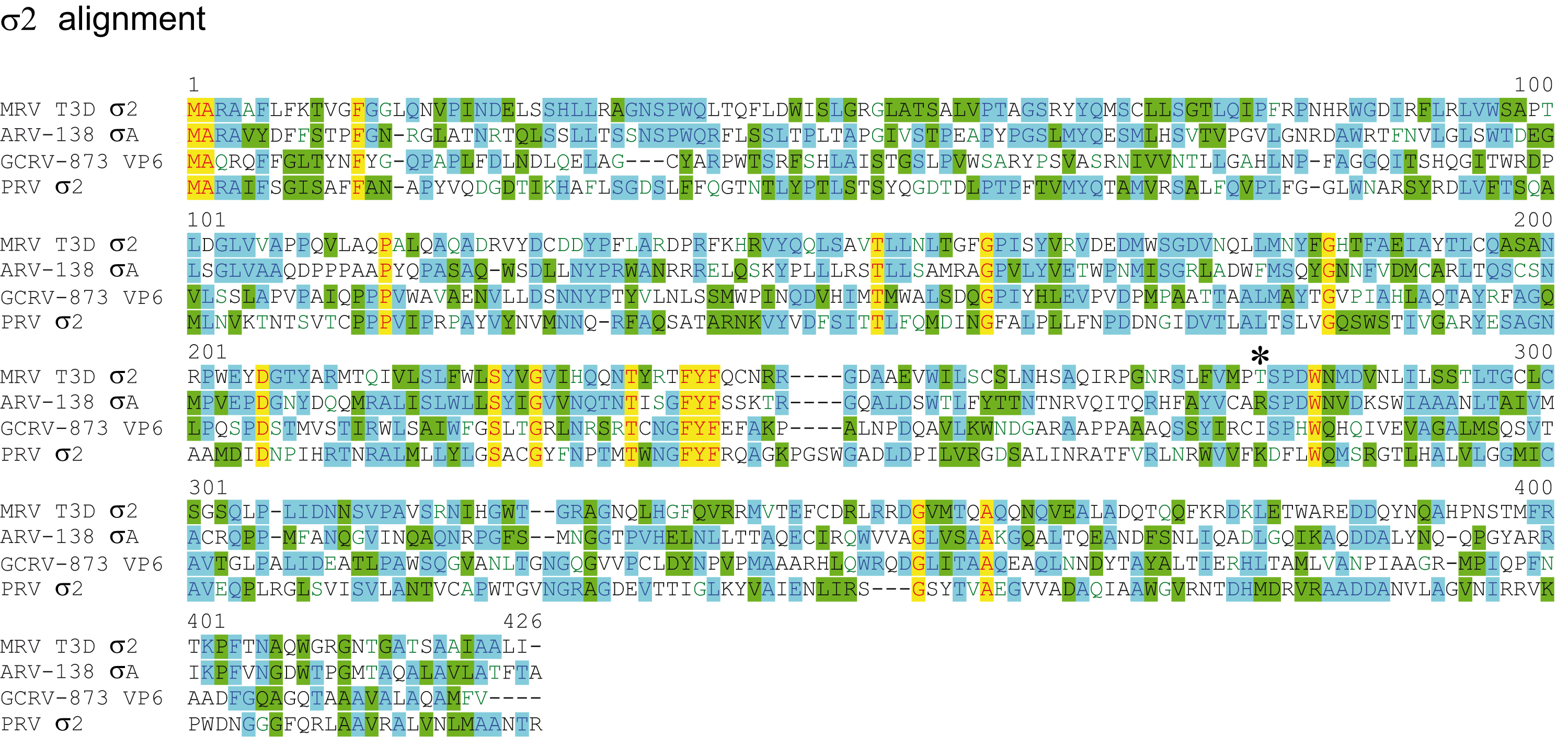

Supplement: Figure S8 — Multiple sequence alignment of PRV S2 ORF encoding the inner capsid σ2 protein with the homologues proteins from the reovirus prototype strains MRV T3D σ2, ARV σA and GCRV-873 VP6. * = R273, one of two arginines in ARV σA linked to dsRNA binding and nucleolar localization, conserved in fusogenic orthoreoviruses. (TIF) [file pone.0070075.s008.tif]

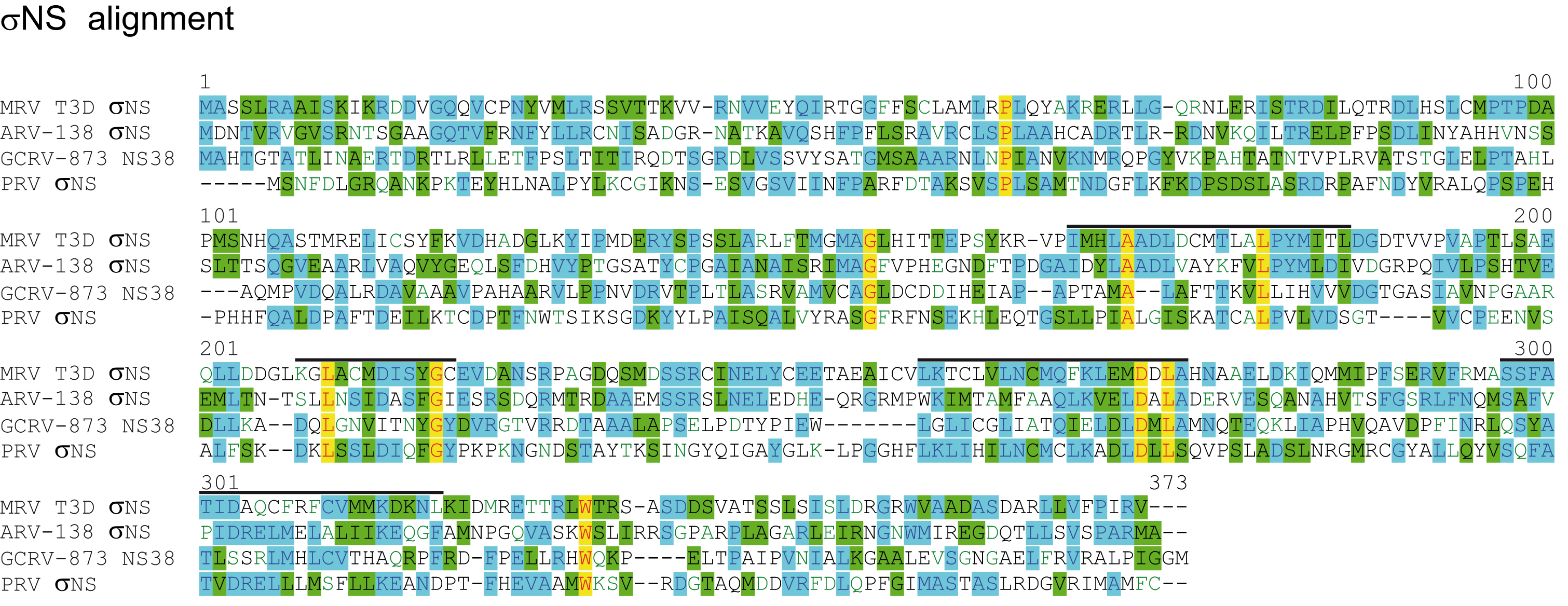

Supplement: Figure S9 — Multiple sequence alignment of PRV S3 ORF encoding the putative σNS protein aligned with σNS/NS38 proteins from reovirus prototype strains MRV T3D, ARV-138 and GCRV-873. Solid black lines represent sequence regions of higher conservation containing putative nuclear export signals. (TIF) [file pone.0070075.s009.tif]

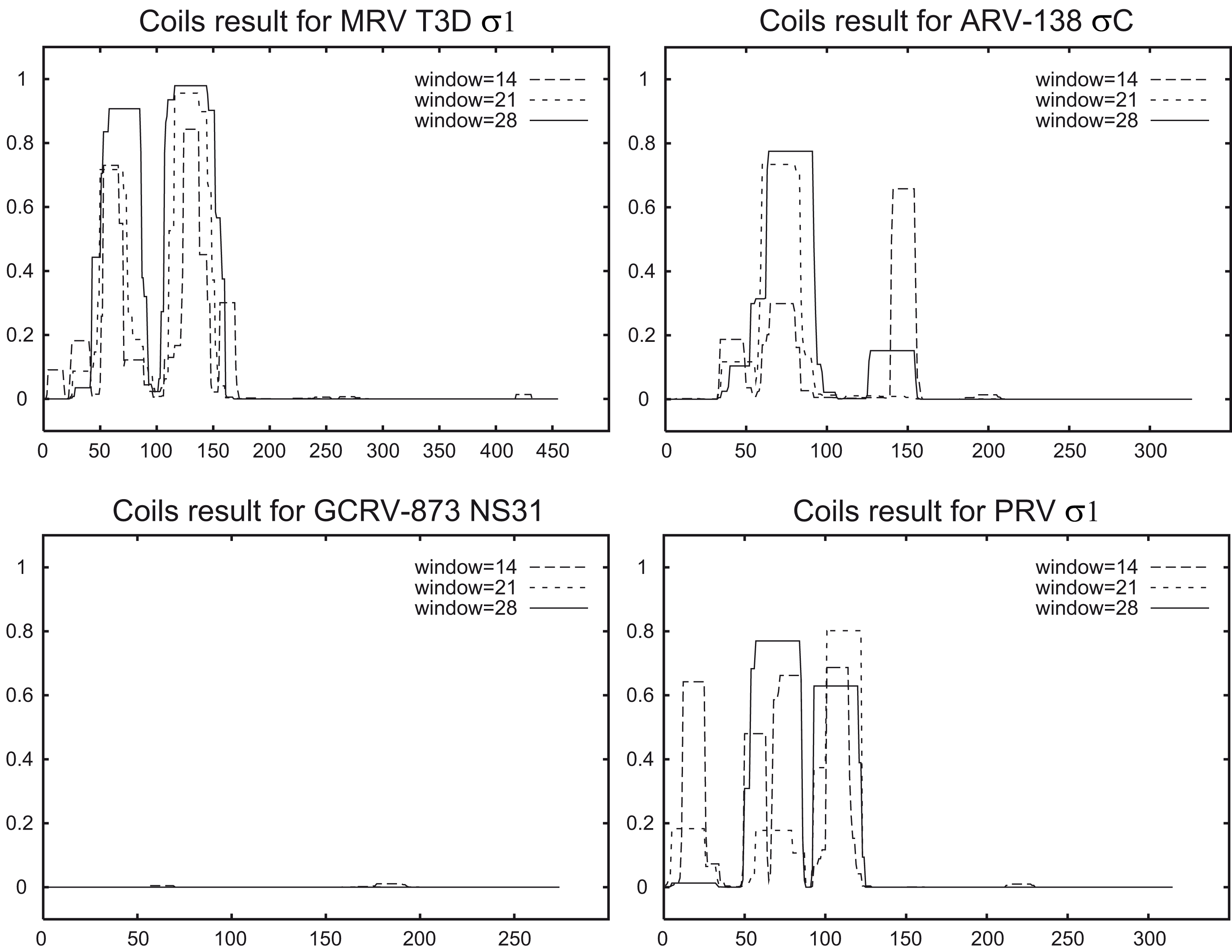

Supplement: Figure S10 — COILS prediction of coiled coil regions in PRV σ1 compared to that of the reovirus prototype strains MRV T3D, ARV-138 and GCRV-873. X-axis displays amino acid positions and the y-axis probabilities. (TIF) [file pone.0070075.s010.tif]

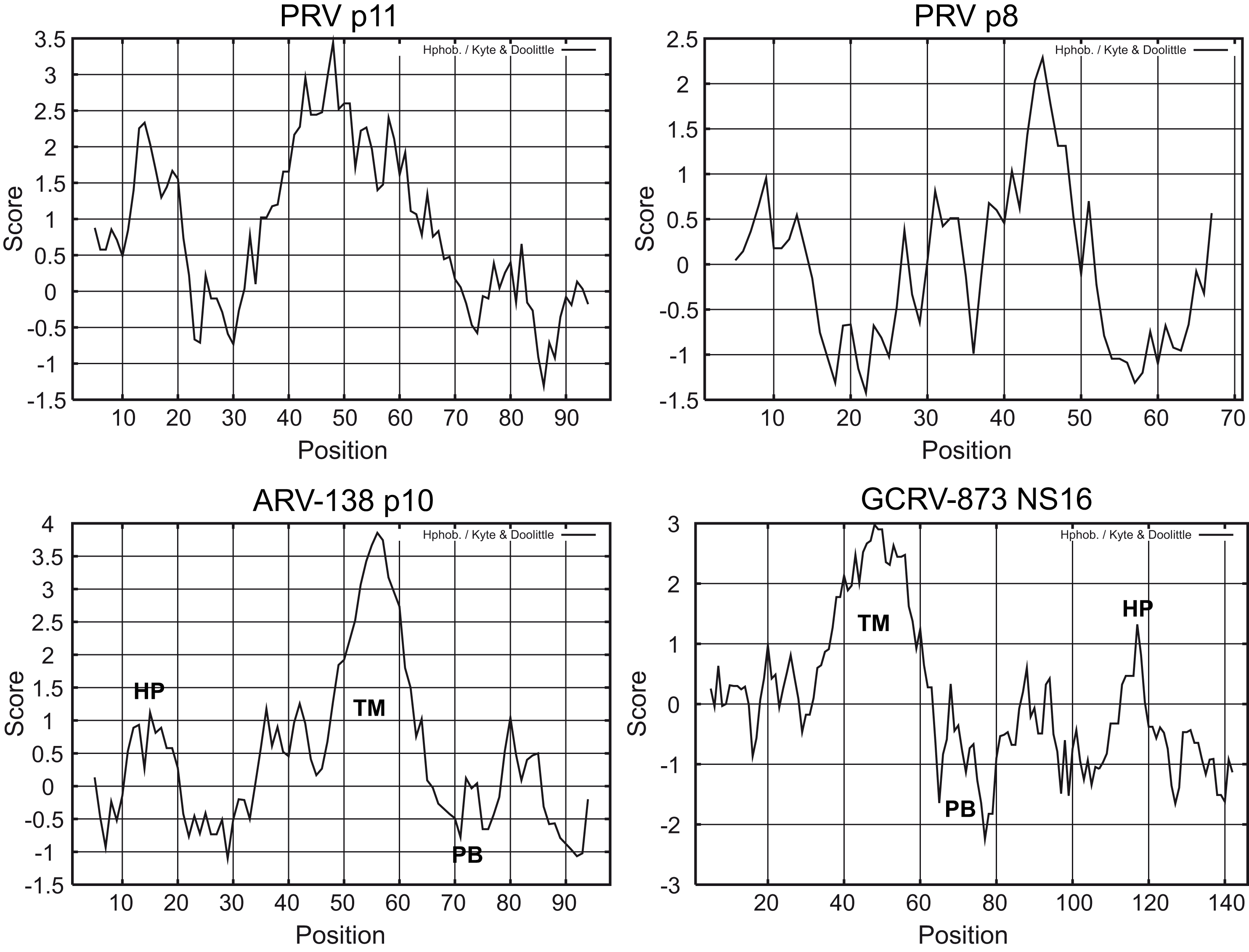

Supplement: Figure S11 — Hydrophobic characters of the hypothetical PRV proteins p11 and p8 as predicted by ProtScale compared to the FAST proteins from ARV-138 and GCRV-873. Predictions were performed using the algorithm by Kyte and Doolittle [149] averaged over a window of nine residues. Positive- and negative scores indicate hydrophobic- and hydrophilic amino acids, respectively. TM = transmembrane domains, PB = polybasic regions and HP = Hydrophobic patch. (TIF) [file pone.0070075.s011.tif]
