# Supplementary material for: Sequence Analysis of the Genome of Piscine Orthoreovirus (PRV) Associated with Heart and Skeletal Muscle Inflammation (HSMI) in Atlantic Salmon (Salmo salar)
Source: PLoS One. 2013 Jul 29;8(7):e70075. doi: 10.1371/journal.pone.0070075 (PMC3726481; doi:10.1371/journal.pone.0070075)
Supplement: Table S1 — Genbank accession numbers for reovirus nucleotide sequences used in the study. (DOCX) [file pone.0070075.s012.docx]

**Table S1:** Genbank accession numbers for reovirus nucleotide sequences used in the study.

| **Reovirus** | **L1** | **L2** | **L3** | **M1** | **M2** | **M3** | **S1** | **S2** | **S3** | **S4** |
| --- | --- | --- | --- | --- | --- | --- | --- | --- | --- | --- |
| **strain** |  |  |  |  |  |  |  |  |  |  |
| PRV | GU994015 | GU994014 | GU994013 | GU994017 | GU994016 | GU994018 | GU994022 | GU994019 | GU994020 | KC915033 |
| MRV T3D^a^ | M31058 | AF378008 | NC004274 | M27261 | M20161 | NC004281 | NC004277 | M25780 | X01627 | NC004276 |
| ARV-138^b^ | EU707935 | EU707937 | EU707933 | AY557188 | AY750052 | AY557190 | AF218359 | AF059717 | AF059725 | AF059721 |
| GCRV-873^c^ | AF260512 | AF260511 | AF260513 | AF403391 | AF403392 | AF403390 | AF403393 | AF403394 | AF403395 | AF403396 |

^a,b,c^Gene segment assignment according to PRV, with the homologues gene segments from MRV, ARV and GCRV. See Table 1 for correct naming of the gene segments for mammalian orthoreoviruses (MRV), the avian orthoreoviruses (ARV) and aquareoviruses (GCRV).
